# Multi-omics single-cell data integration and regulatory inference with graph-linked embedding

**DOI:** 10.1038/s41587-022-01284-4

**Published:** 2022-05-02

**Authors:** Zhi-Jie Cao, Ge Gao

**Affiliations:** 1 State Key Laboratory of Protein and Plant Gene Research, School of Life Sciences, Biomedical Pioneering Innovative Center (BIOPIC) and Beijing Advanced Innovation Center for Genomics (ICG), Center for Bioinformatics (CBI), Peking University, Beijing, China; 2 Changping Laboratory, Beijing, China

**Keywords:** Data integration, Machine learning, Gene regulation, Software

## Abstract

Despite the emergence of experimental methods for simultaneous measurement of multiple omics modalities in single cells, most single-cell datasets include only one modality. A major obstacle in integrating omics data from multiple modalities is that different omics layers typically have distinct feature spaces. Here, we propose a computational framework called GLUE (graph-linked unified embedding), which bridges the gap by modeling regulatory interactions across omics layers explicitly. Systematic benchmarking demonstrated that GLUE is more accurate, robust and scalable than state-of-the-art tools for heterogeneous single-cell multi-omics data. We applied GLUE to various challenging tasks, including triple-omics integration, integrative regulatory inference and multi-omics human cell atlas construction over millions of cells, where GLUE was able to correct previous annotations. GLUE features a modular design that can be flexibly extended and enhanced for new analysis tasks. The full package is available online at https://github.com/gao-lab/GLUE.

## Abstract (web summary)

Different single-cell data modalities are integrated at atlas-scale by modeling regulatory interactions.

## Main

Recent technological advances in single-cell sequencing have enabled the probing of regulatory maps through multiple omics layers, such as chromatin accessibility (single-cell ATAC-sequencing (scATAC-seq)^1,2^), DNA methylation (snmC-seq^3^, sci-MET^4^) and the transcriptome (scRNA-seq^5,6^), offering a unique opportunity to unveil the underlying regulatory bases for the functionalities of diverse cell types^7^. While simultaneous assays have recently emerged^8–11^, different omics are usually measured independently and produce unpaired data, which calls for effective and efficient in silico multi-omics integration^12,13^.

Computationally, one major obstacle faced when integrating unpaired multi-omics data (also known as diagonal integration) is the distinct feature spaces of different modalities (for example, accessible chromatin regions in scATAC-seq versus genes in scRNA-seq)^14^. A quick fix is to convert multimodality data into one common feature space based on prior knowledge and apply single-omics data integration methods^15–18^. Such explicit ‘feature conversion’ is straightforward, but has been reported to result in information loss^19^. Algorithms based on coupled matrix factorization circumvent explicit conversion but hardly handle more than two omics layers^20,21^. An alternative option is to match cells from different omics layers via nonlinear manifold alignment, which removes the requirement of prior knowledge completely and could reduce inter-modality information loss in theory^22–25^; however, this technique has mostly been applied to relatively small datasets with limited number of cell types.

The ever-increasing volume of data is another serious challenge^26^. Recently developed technologies can routinely generate datasets at the scale of millions of cells^27–29^, whereas current integration methods have only been applied to datasets with much smaller volumes^15,17,20–23^. To catch up with the growth in data throughput, computational integration methods should be designed with scalability in mind.

Hereby, we introduce GLUE (graph-linked unified embedding), a modular framework for integrating unpaired single-cell multi-omics data and inferring regulatory interactions simultaneously. By modeling the regulatory interactions across omics layers explicitly, GLUE bridges the gaps between various omics-specific feature spaces in a biologically intuitive manner. Systematic benchmarks and case studies demonstrate that GLUE is accurate, robust and scalable for heterogeneous single-cell multi-omics data. Furthermore, GLUE is designed as a generalizable framework that allows for easy extension and quick adoption to particular scenarios in a modular manner. GLUE is publicly accessible at https://github.com/gao-lab/GLUE.

## Results

### Unpaired multi-omics integration via graph-guided embeddings

**Fig. 1:**
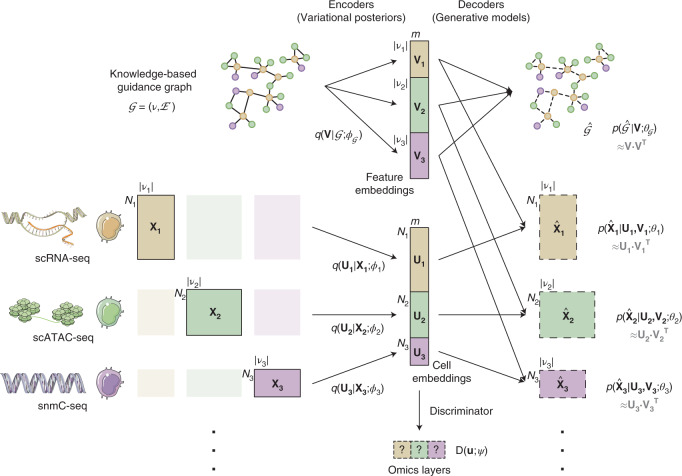
Architecture of the GLUE framework. Denoting unpaired data from three omics layer as ${{{\mathbf{X}}}}_1 \in {\Bbb R}^{N_1 \times \left| {{{{\mathcal{V}}}}_1} \right|},{{{\mathbf{X}}}}_2 \in {\Bbb R}^{N_2 \times \left| {{{{\mathcal{V}}}}_2} \right|},{{{\mathbf{X}}}}_3 \in {\Bbb R}^{N_3 \times \left| {{{{\mathcal{V}}}}_3} \right|}$, where *N*_1_, *N*_2_, *N*_3_ are cell numbers, and ${{{\mathcal{V}}}}_1,{{{\mathcal{V}}}}_2,{{{\mathcal{V}}}}_3$ are sets of omics features in each layer, GLUE uses omics-specific variational autoencoders to learn low-dimensional cell embeddings **U**_1_, **U**_2_, **U**_3_ from each omics layer. The data dimensionality and generative distribution can differ across layers, but the embedding dimension *m* is shared. To link the omics-specific data spaces, GLUE makes use of prior knowledge about regulatory interactions in the form of a guidance graph ${{{\mathcal{G}}}} = \left( {{{{\mathcal{V}}}},{{{\mathcal{E}}}}} \right)$, where vertices ${{{\mathcal{V}}}} = {{{\mathcal{V}}}}_1 \cup {{{\mathcal{V}}}}_2 \cup {{{\mathcal{V}}}}_3$ are omics features. A graph variational autoencoder is used to learn feature embeddings ${{{\mathbf{V}}}} = \left( {{{{\mathbf{V}}}}_1^ \top ,{{{\mathbf{V}}}}_2^ \top ,{{{\mathbf{V}}}}_3^ \top } \right)^ \top$ from the prior knowledge-based guidance graph, which are then used in data decoders to reconstruct omics data via inner product with cell embeddings, effectively linking the omics-specific data spaces to ensure a consistent embedding orientation. Last, an omics discriminator D is used to align the cell embeddings of different omics layers via adversarial learning. $\phi _1,\phi _2,\phi _3,\phi _{{{\mathcal{G}}}}$ represent learnable parameters in data and graph encoders. $\theta _1,\theta _2,\theta _3,\theta _{{{\mathcal{G}}}}$ represent learnable parameters in data and graph decoders. *ψ* represents learnable parameters in the omics discriminator.

Inspired by previous studies, we model cell states as low-dimensional cell embeddings learned through variational autoencoders^30,31^. Given their intrinsic differences in biological nature and assay technology, each omics layer is equipped with a separate autoencoder that uses a probabilistic generative model tailored to the layer-specific feature space (Fig. 1 and Methods).

Taking advantage of prior biological knowledge, we propose the use of a knowledge-based graph (‘guidance graph’) that explicitly models cross-layer regulatory interactions for linking layer-specific feature spaces; the vertices in the graph correspond to the features of different omics layers, and edges represent signed regulatory interactions. For example, when integrating scRNA-seq and scATAC-seq data, the vertices are genes and accessible chromatin regions (that is, ATAC peaks), and a positive edge can be connected between an accessible region and its putative downstream gene. Then, adversarial multimodal alignment of the cells is performed as an iterative optimization procedure, guided by feature embeddings encoded from the graph^32^ (Fig. 1 and Methods). Notably, when the iterative process converges, the graph can be refined with inputs from the alignment procedure and used for data-oriented regulatory inference (see below for more details).

### Systematic benchmarking demonstrates superior performance

We first benchmarked GLUE against multiple popular unpaired multi-omics integration methods^15–18,23–25,33^ using three gold-standard datasets generated by recent simultaneous scRNA-seq and scATAC-seq technologies (SNARE-seq^8^, SHARE-seq^9^ and 10X Multiome^34^), along with two unpaired datasets (Nephron^35^ and MOp^36^).

**Fig. 2:**
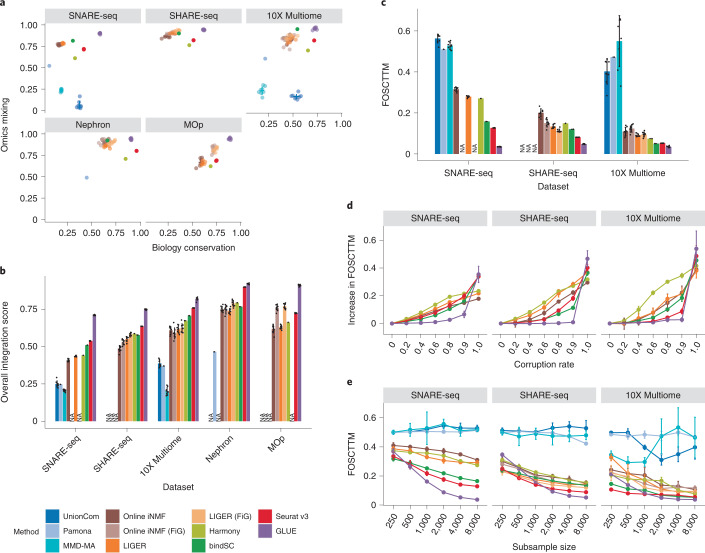
Systematic benchmarks of integration performance. **a**, Biological conservation score versus omics integration score for different integration methods. **b**, Overall integration score (defined as 0.6 × biology conservation + 0.4 × omics integration) of different integration methods (*n* = 8 repeats with different model random seeds). **c**, Single-cell level alignment error (quantified by FOSCTTM) of different integration methods (*n* = 8 repeats with different model random seeds). **d**, Increases in FOSCTTM at different prior knowledge corruption rates for integration methods that rely on prior feature relations (*n* = 8 repeats with different corruption random seeds). **e**, FOSCTTM values of different integration methods on subsampled datasets of varying sizes (*n* = 8 repeats with different subsampling random seeds). FiG is an alternative feature conversion method recommended by online iNMF and LIGER (Methods). Online iNMF and LIGER could not run with FiG conversion on the SNARE-seq data because the raw ATAC fragment file was not available, thus marked as ‘NA’. Other NA marks were made because of memory overflow. The error bars indicate mean ± s.d.

**Extended Data Fig. 1:**
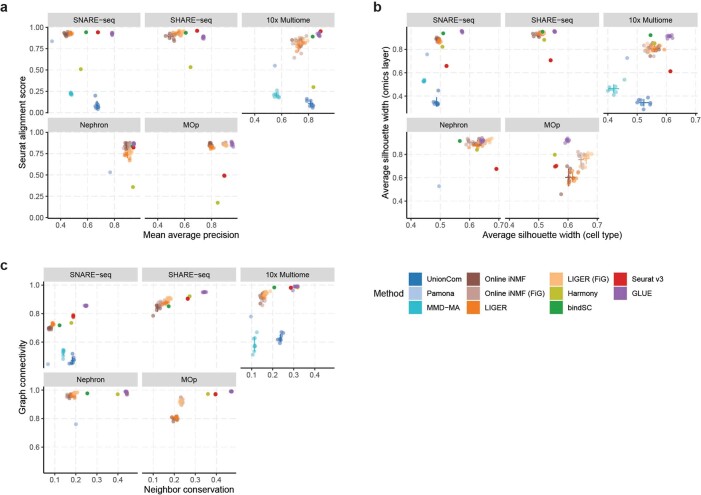
Individual metrics for evaluating integration performance. **a**, Mean average precision vs. Seurat alignment score for different integration methods. Higher mean average precision indicates higher cell type resolution, and higher Seurat alignment score indicates better omics mixing. **b**, Cell type vs. omics layer average silhouette width for different integration methods. Higher cell type average silhouette width indicates higher cell type resolution, and higher omics layer average silhouette width indicates better omics mixing. **c**, Neighbor conservation vs. graph connectivity for different integration methods. Higher neighbor conservation indicates better conservation of manifold structure in each original layer, and higher graph connectivity indicates better omics mixing. n=8 repeats with different model random seeds. The error bars indicate mean ± s.d.

An effective integration method should match the corresponding cell states from different omics layers, producing cell embeddings where the biological variation is faithfully conserved and the omics layers are well mixed. Compared to other methods, GLUE achieved high level of biology conservation and omics mixing simultaneously (Fig. 2a, each quantified by three separate metrics as shown in Extended Data Fig. 1), and was consistently the best method across all benchmark datasets in terms of overall score (Fig. 2b, see Methods for details on metric aggregation); these results were also validated by uniform manifold approximation and projection (UMAP) visualization of the aligned cell embeddings (Supplementary Figs. 1–5).

An optimal integration method should produce accurate alignments not only at the cell type level but also at finer scales. Exploiting the ground truth cell-to-cell correspondence in the gold-standard datasets, we further quantified single-cell level alignment error via the FOSCTTM (fraction of samples closer than the true match) metric^25^. On all three datasets, GLUE achieved the lowest FOSCTTM, decreasing the alignment error by large margins compared to the second-best method on each dataset (Fig. 2c, the decreases were 3.6-fold for SNARE-seq, 1.7-fold for SHARE-seq and 1.5-fold for 10X Multiome).

**Extended Data Fig. 2:**
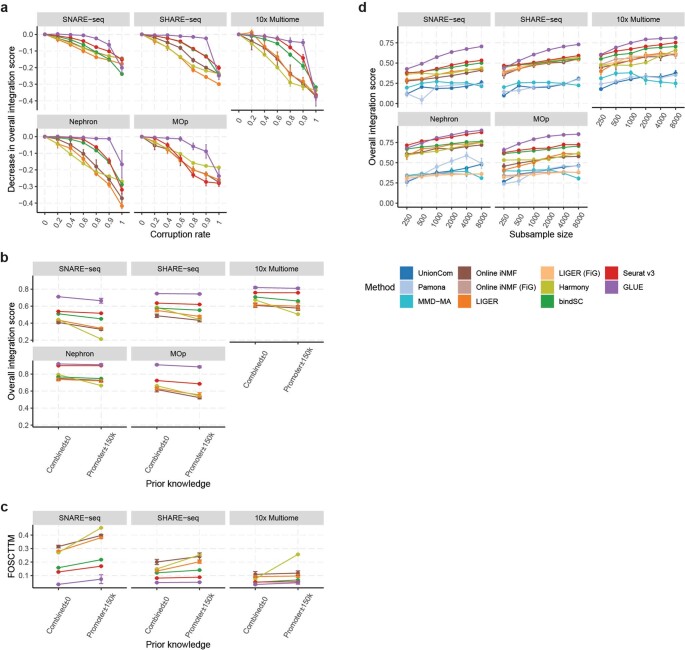
Effect of prior knowledge and data size on integration performance. **a**, Decrease in overall integration score at different prior knowledge corruption rates for integration methods that rely on prior feature relations (n=8 repeats with different corruption random seeds). **b**, Overall integration score, and **c**, FOSCTTM with different schemes of connecting peaks and genes as prior regulatory knowledge, for integration methods that rely on prior feature relations (n=8 repeats with different model random seeds). ‘Combined±0’ is the standard scheme where peaks overlapping gene body or promoter regions are linked. ‘Promoter±150k’ means that peaks are linked to genes if they locate within 150kb from the gene promoter, weighted by a power-law function that models chromatin contact probability^42,43^. **d**, Overall integration score of different integration methods on subsampled datasets of varying sizes (n=8 repeats with different subsampling random seeds). The error bars indicate mean ± s.d.

During the evaluation described above, we adopted a standard schema (ATAC peaks were linked to RNA genes if they overlapped in the gene body or proximal promoter regions) to construct the guidance graph for GLUE and to perform feature conversion for other conversion-based methods. Given that our current knowledge about the regulatory interactions is still far from prefect, a useful integration method must be robust to such inaccuracies. Thus, we further assessed the methods’ robustness to corruption of regulatory interactions by randomly replacing varying fractions of existing interactions with nonexistent ones. For all three datasets, GLUE exhibited the smallest performance changes even at corruption rates as high as 90% (Fig. 2d and Extended Data Fig. 2a), suggesting its superior robustness. Consistently, we found that using alternative guidance graphs defined in larger genomic windows had minimal influence on integration performance (Extended Data Fig. 2b,c).

**Extended Data Fig. 3:**
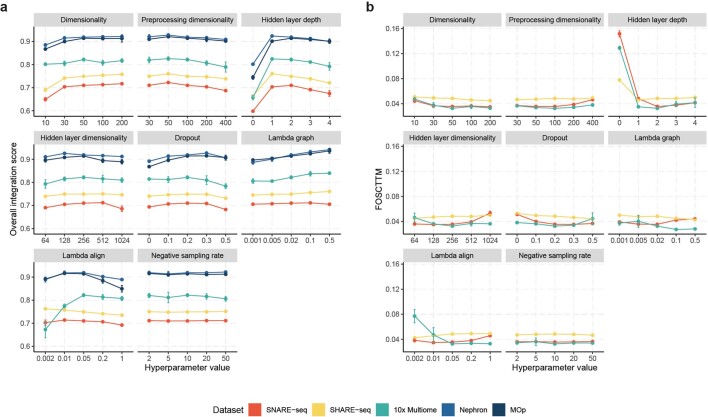
Integration performance of GLUE under different hyperparameter settings. Integration performance is quantified by **a**, overall integration score, and **b**, FOSCTTM (n=4 repeats with different model random seeds). The error bars indicate mean ± s.d. ‘Dimensionality’ denotes the cell embedding dimensionality. ‘Preprocessing dimensionality’ is the reduced dimensionality used for the first transformation layers of the data encoders (see Methods). ‘Hidden layer depth’ is the number of hidden layers in the data encoders and modality discriminator. ‘Hidden layer dimensionality’ is the dimensionality of hidden layers in the data encoders and modality discriminator. ‘Dropout’ is the dropout rate of hidden layers in data encoders and modality discriminator. ‘Lambda graph’ is the weight of the graph loss ($\lambda _{{{\mathcal{G}}}}$). ‘Lambda align’ is the weight of the adversarial alignment (*λ*_D_). ‘Negative sampling rate’ is the number of empirical samples used in negative edge sampling (samples from *p*_ns_). For each hyperparameter, the center value is the default. To control computational cost, one hyperparameter was varied at a time, with all others set to their default values. The performance of GLUE was robust across a wide range of hyperparameter settings, except for failed alignments in which the adversarial alignment weight was too low or no hidden layers were used in the neural networks (equivalently a linear model with insufficient capacity).

**Extended Data Fig. 4:**
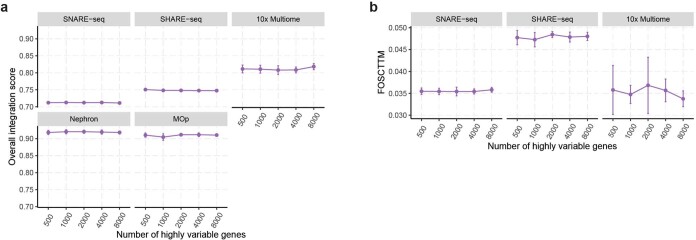
Integration performance of GLUE with different numbers of highly variable genes. Integration performance is quantified by **a**, overall integration score, and **b**, FOSCTTM (n=8 repeats with different model random seeds). The error bars indicate mean ± s.d.

**Extended Data Fig. 5:**
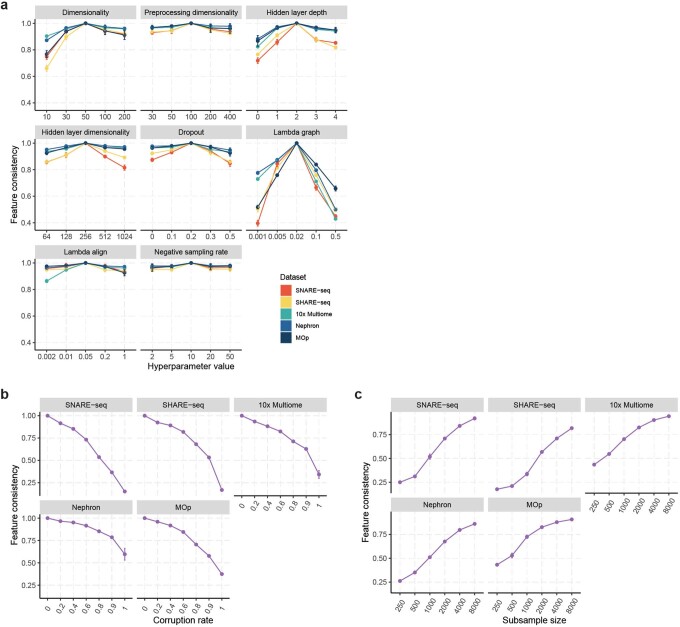
Robustness of GLUE feature embeddings. Consistency of feature embeddings as defined by the conservation of feature-feature cosine similarity (Methods), under **a**, different hyperparameter settings (n=4 repeats with different model random seeds), **b**, different prior knowledge corruption rates (n=8 repeats with different corruption random seeds), and **c**, different number of subsampled cells (n=8 repeats with different subsampling random seeds). The error bars indicate mean ± s.d. Feature embeddings are robust across all hyperparameters except for $\lambda _{{{\mathcal{G}}}}$, which directly controls the contribution of guidance graph. Consistency also remains high (> 0.8) with up to 40% of prior knowledge corrupted, and a minimal of ~4,000 subsampled cells.

Given its neural network-based nature, GLUE may suffer from undertraining when working with small datasets. Thus, we repeated the evaluations using subsampled datasets of various sizes. GLUE remained the top-ranking method with as few as 2,000 cells, but the alignment error increased more steeply when the data volume decreased to less than 1,000 cells (Fig. 2e and Extended Data Fig. 2d). Additionally, we also noted that the integration performance of GLUE was robust for a wide range of hyperparameter and feature selection settings (Extended Data Figs. 3 and 4). Apart from the cell embeddings, the feature embeddings of GLUE also exhibit considerable robustness to hyperparameter settings, prior knowledge corruption and data subsampling (Extended Data Fig. 5).

**Extended Data Fig. 6:**
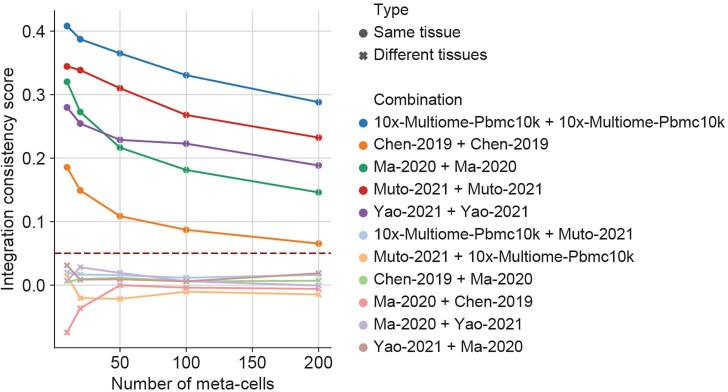
Integration consistency score for detecting over-correction. Integration consistency scores with varying numbers of meta-cells for different dataset combinations. Same-tissue combinations represent proper correction, and different-tissue combinations represent over-correction. Dashed horizontal line indicate integration consistency score = 0.05.

In addition to the systematical difference among omics layers, single-cell data are often complicated by batch effect within the same layer. For example, the SHARE-seq data was processed in four libraries, one of which showed batch effect compared to the other three in scRNA-seq (Supplementary Fig. 6a), while the Nephron data profiled four donors, all of which showed substantial batch effect against each other in both scRNA-seq and scATAC-seq (Supplementary Fig. 7a,c). As a solution to such complex scenarios, GLUE provides batch correction capability by including batch as a decoder covariate (Methods). With batch correction enabled, GLUE was able to correct for these batch effects effectively, producing substantially better batch mixing (Supplementary Fig. 6b and Supplementary Fig. 7b,d). To guard against potential over-correction, for example, when forcing an integration over datasets lacking common cell states, we devised a diagnostic metric called the integration consistency score, which measures the consistency between the integrated multi-omics space and prior knowledge in the guidance graph (Methods). We observed substantially lower scores (close to 0) when integrating data from inconsistent tissues compared to integrating within the same tissue, making it a reliable indicator of integration quality (Extended Data Fig. 6).

### GLUE enables effective triple-omics integration

Benefitting from a modular design and scalable adversarial alignment, GLUE readily extends to more than two omics layers. As a case study, we used GLUE to integrate three distinct omics layers of neuronal cells in the adult mouse cortex, including gene expression^37^, chromatin accessibility^38^ and DNA methylation^3^.

Unlike chromatin accessibility, gene body DNA methylation generally shows a negative correlation with gene expression in neuronal cells^39^. GLUE natively supports the mixture of regulatory effects by modeling edge signs in the guidance graph. Such a strategy avoids data inversion, which is required by previous methods^16,17^ and can break data sparsity and the underlying distribution. For the triple-omics guidance graph, we linked gene body mCH and mCG levels to genes via negative edges, while the positive edges between accessible regions and genes remained the same.

**Fig. 3:**
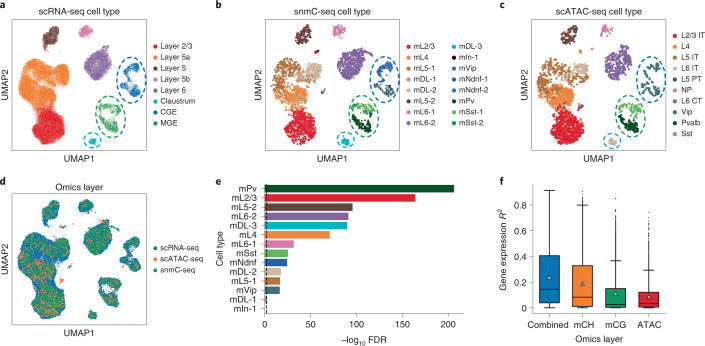
Triple-omics integration of the mouse cortex. **a**–**c**, UMAP visualizations of the integrated cell embeddings for scRNA-seq (**a**), snmC-seq (**b**) and scATAC-seq (**c**), colored by the original cell types. Cells aligning with ‘mPv’ and ‘mSst’ are highlighted with green circles. Cells aligning with ‘mNdnf’ and ‘mVip’ are highlighted with dark blue circles. Cells aligning with ‘mDL-3’ are highlighted with light blue circles. **d**, UMAP visualizations of the integrated cell embeddings for all cells, colored by omics layers. **e**, Significance of marker gene overlap for each cell type across all three omics layers (three-way Fisher’s exact test^40^). The dashed vertical line indicates that FDR = 0.01. We observed highly significant marker overlap (FDR < 5 × 10^−17^) for 12 out of the 14 cell types, indicating reliable alignment. For the remaining two cell types, ‘mDL-1’ had marginally significant marker overlap with FDR = 0.003, while the ‘mIn-1’ cells in snmC-seq did not properly align with the scRNA-seq or scATAC-seq cells. **f**, Coefficient of determination (*R*^2^) for predicting gene expression based on each epigenetic layer as well as the combination of all layers (*n* = 2,677 highly variable genes common to all three omics layers). The box plots indicate the medians (centerlines), means (triangles), first and third quartiles (bounds of boxes) and 1.5× interquartile range (whiskers).

The GLUE alignment successfully revealed a shared manifold of cell states across the three omics layers (Fig. 3a–d). Notably, the original cell types were not annotated at the same resolution, and many could be further clustered into smaller subtypes even within single layers (Supplementary Fig. 8a–f). To unify the cell type annotations, neighbor-based label transfer was conducted using the integrated cell embeddings and we observed highly significant marker overlap (Fig. 3e, three-way Fisher’s exact test^40^, false discovery rate (FDR) < 5 × 10^−17^) for 12 out of the 14 mapped cell types (Supplementary Figs. 8g–o and 9 and Methods), indicating reliable alignment. The GLUE alignment helped improve the effects of cell typing in all omics layers, including the further partitioning of the scRNA-seq ‘MGE’ cluster into *Pvalb*^*+*^ (‘mPv’) and *Sst*^*+*^ (‘mSst’) subtypes (highlighted with green circles/flows in Fig. 3 and Supplementary Fig. 8), the partitioning of the scRNA-seq ‘CGE’ cluster and scATAC-seq ‘Vip’ cluster into *Vip*^*+*^ (‘mVip’) and *Ndnf*^*+*^ (‘mNdnf’) subtypes (highlighted with dark blue circles/flows in Fig. 3 and Supplementary Fig. 8), and the identification of snmC-seq ‘mDL-3’ cells and a subset of scATAC-seq ‘L6 IT’ cells as claustrum cells (highlighted with light blue circles/flows in Fig. 3 and Supplementary Fig. 8).

Such triple-omics integration also sheds light on the quantitative contributions of different epigenetic regulation mechanisms (Methods). Among mCH, mCG and chromatin accessibility, we found that the mCH level had the highest predictive power for gene expression in cortical neurons (average *R*^2^ = 0.187). When all epigenetic layers were considered, the expression predictability increased further (average *R*^2^ = 0.236), suggesting the presence of nonredundant contributions (Fig. 3f). Among the neurons of different layers, DNA methylation (especially mCH) exhibited slightly higher predictability for gene expression in deeper layers than in superficial layers (Supplementary Fig. 10a). Across all genes, the predictability of gene expression was generally correlated among the different epigenetic layers (Supplementary Fig. 10b). We also observed varying associations with gene characteristics. For example, mCH had higher expression predictability for longer genes, which was consistent with previous studies^17,41^, while chromatin accessibility contributed more to genes with higher expression variability (Supplementary Fig. 10c). We also repeated the same analysis using online iNMF, which is currently the only other method capable of integrating the three omics layers simultaneously, but it produced much lower cell type resolution and epigenetic correlation (Supplementary Fig. 11).

### Integrative regulatory inference with GLUE

The incorporation of a graph explicitly modeling regulatory interactions in GLUE further enables a Bayesian-like approach that combines prior knowledge and observed data for posterior regulatory inference. Specifically, since the feature embeddings are designed to reconstruct the knowledge-based guidance graph and single-cell multi-omics data simultaneously (Fig. 1), their cosine similarities should reflect information from both aspects, which we adopt as ‘regulatory scores’.

**Fig. 4:**
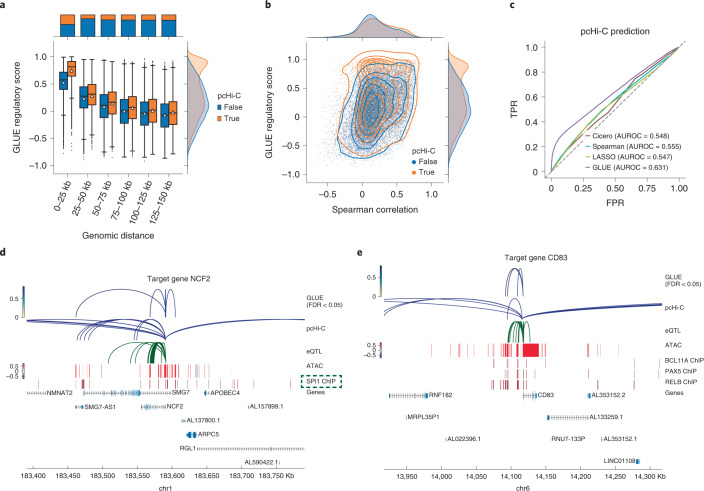
Integrative regulatory inference in peripheral blood mononuclear cells. **a**, GLUE regulatory scores for peak–gene pairs across different genomic ranges, grouped by whether they had pcHi-C support. The box plots indicate the medians (centerlines), means (triangles), first and third quartiles (bounds of boxes) and 1.5× interquartile range (whiskers). **b**, Comparison between the GLUE regulatory scores and the empirical peak–gene correlations computed on paired cells. Peak–gene pairs are colored by whether they had pcHi-C support. **c**, Receiver operating characteristic curves for predicting pcHi-C interactions based on different peak–gene association scores. AUROC is the area under the receiver operating characteristic curve. **d**,**e**, GLUE-identified *cis*-regulatory interactions of *NCF2* (**d**) and *CD83* (**e**), along with individual regulatory evidence. SPI1 (highlighted with a green box) is a known regulator of *NCF2*.

As a demonstration, we used the official peripheral blood mononuclear cell Multiome dataset from 10X^34^ and fed it to GLUE as unpaired scRNA-seq and scATAC-seq data. To capture remote *cis*-regulatory interactions, we used a long-range guidance graph connecting ATAC peaks and RNA genes in 150-kb windows weighted by a power-law function that models chromatin contact probability^42,43^ (Methods). Visualization of cell embeddings confirmed that the GLUE alignment was correct and accurate (Supplementary Fig. 12a,b). As expected, we found that the regulatory score was negatively correlated with genomic distance (Fig. 4a) and positively correlated with the empirical peak–gene correlation (computed with paired cells, Fig. 4b), with robustness across different random seeds (Supplementary Fig. 12c).

To further assess whether the score reflected actual *cis*-regulatory interactions, we compared it with external evidence, including pcHi-C^44^ and eQTL^45^. The GLUE regulatory score was higher for pcHi-C-supported peak–gene pairs in all distance ranges (Fig. 4a) and was a better predictor of pcHi-C interactions than empirical peak–gene correlations (Fig. 4b), as well as LASSO and Cicero^43^, the coaccessibility-based regulatory prediction method (Fig. 4c and Supplementary Fig. 12d). The same held for eQTL (Supplementary Fig. 12e–h).

The GLUE framework also allows additional regulatory evidence, such as pcHi-C, to be incorporated intuitively via the guidance graph. Thus, we further trained models with a composite guidance graph containing distance-weighted interactions as well as pcHi-C- and eQTL-supported interactions (Supplementary Fig. 13). The significance of regulatory score was evaluated by comparing it to a NULL distribution obtained from randomly shuffled feature embeddings (Methods). As expected, while the multi-omics alignment was insensitive to the change in guidance graph, the inferred regulatory interactions showed stronger enrichment for pcHi-C and eQTL (Supplementary Fig. 13a–d). Large fractions of high-confidence interactions simultaneously supported by pcHi-C, eQTL and correlation could be robustly recovered (FDR < 0.05), even if they were corrupted in the guidance graph (Supplementary Fig. 13e). Furthermore, the GLUE-derived transcription factor (TF-)target gene network (Methods) showed more significant agreement with manually curated connections in the TRRUST v2 database^46^ than individual evidence-based networks (Supplementary Figs. 13f and Supplementary Fig. 14 and Supplementary Data 2).

We noticed that the GLUE-inferred *cis*-regulatory interactions could provide hints about the regulatory mechanisms of known TF-target pairs. For example, SPI1 is a known regulator of the *NCF2* gene, and both are highly expressed in monocytes (Supplementary Fig. 15a,b). GLUE identified three remote regulatory peaks for *NCF2* with various pieces of evidence, that is, roughly 120 kb downstream, 25 kb downstream and 20 kb upstream from the transcription start site (TSS) (Fig. 4d), all of which were bound by SPI1. Meanwhile, most putative regulatory interactions were previously unknown. For example, *CD83* was linked with three regulatory peaks (two roughly 25 kb upstream, one about 10 kb upstream from the TSS), which were enriched for the binding of three TFs (BCL11A, PAX5 and RELB; Fig. 4e). While *CD83* was highly expressed in both monocytes and B cells, the inferred TFs showed more constrained expression patterns (Supplementary Fig. 15c–f), suggesting that its active regulators might differ per cell type. Supplementary Fig. 16 shows more examples of GLUE-inferred regulatory interactions.

### Atlas-scale integration over millions of cells with GLUE

As technologies continue to evolve, the throughput of single-cell experiments is constantly increasing. Recent studies have generated human cell atlases for gene expression^28^ and chromatin accessibility^29^ containing millions of cells. The integration of these atlases poses a substantial challenge to computational methods due to the sheer volume of data, extensive heterogeneity, low coverage per cell and unbalanced cell type compositions, and has yet to be accomplished at the single-cell level.

**Fig. 5:**
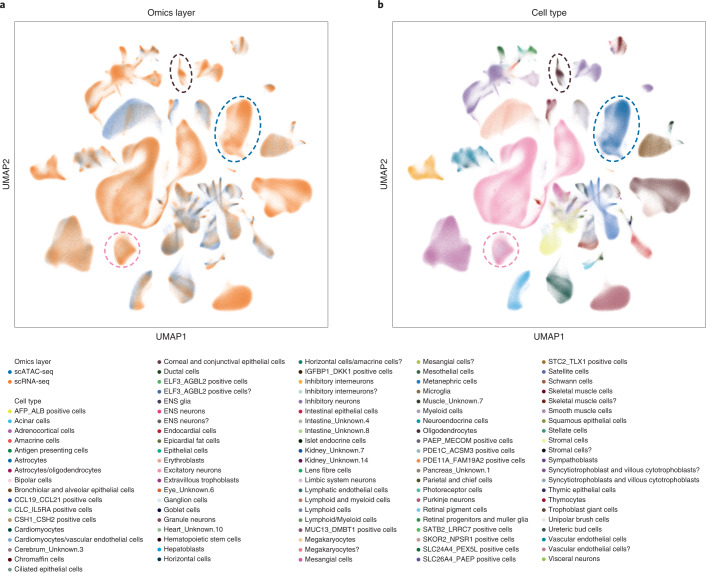
Integration of a multi-omics human cell atlas. **a**,**b**, UMAP visualizations of the integrated cell embeddings, colored by omics layers (**a**) and cell types (**b**). The pink circles highlight cells labeled as ‘Excitatory neurons’ in scRNA-seq but ‘Astrocytes’ in scATAC-seq. The blue circles highlight cells labeled as ‘Astrocytes’ in scRNA-seq but ‘Astrocytes/oligodendrocytes’ in scATAC-seq. The brown circles highlight cells labeled as ‘Oligodendrocytes’ in scRNA-seq but ‘Astrocytes/oligodendrocytes’ in scATAC-seq.

Implemented as a neural network with minibatch optimization, GLUE delivers superior scalability with a sublinear time cost, promising its applicability at the atlas-scale (Supplementary Fig. 17a). Using an efficient multistage training strategy for GLUE (Methods), we successfully integrated the gene expression and chromatin accessibility data into a unified multi-omics human cell atlas (Fig. 5).

While the aligned atlas was largely consistent with the original annotations^29^ (Supplementary Fig. 17c–e), we also noticed several discrepancies. For example, cells originally annotated as ‘Astrocytes’ in scATAC-seq were aligned to an ‘Excitatory neurons’ cluster in scRNA-seq (highlighted with pink circles/flows in Supplementary Fig. 17). Further inspection revealed that canonical radial glial markers such as *PAX6*, *HES1* and *HOPX*^47,48^ were actively transcribed in this cluster, both in the RNA and ATAC domain (Supplementary Fig. 18), with chromatin priming^9^ also detected at both neuronal and glial markers (Supplementary Figs. 19–21), suggesting that the cluster consists of multipotent neural progenitors (likely radial glial markers) rather than excitatory neurons or astrocytes as originally annotated. GLUE-based integration also resolved several scATAC-seq clusters that were ambiguously annotated. For example, the ‘Astrocytes/Oligodendrocytes’ cluster was split into two halves and aligned to the ‘Astrocytes’ and ‘Oligodendrocytes’ clusters of scRNA-seq (highlighted, respectively, with blue and brown circles/flows in Supplementary Fig. 17), which was also supported by marker expression and accessibility (Supplementary Figs. 20 and 21). These results demonstrate the unique value of atlas-scale multi-omics integration where cell typing can be done in an unbiased, data-oriented manner across modalities without losing single-cell resolution. In particular, the incorporation of batch correction could further enable effective curation of new datasets with the integrated atlas as a global reference^49^.

In comparison, we also attempted to perform integration using online iNMF, which was the only other method capable of integrating the data at full scale, but the result was far from optimal (Supplementary Figs. 22a,b and 23). Meanwhile, an attempt to integrate the data as aggregated metacells (Methods) via the popular Seurat v3 method also failed (Supplementary Fig. 22c,d).

## Discussion

Combining omics-specific autoencoders with graph-based coupling and adversarial alignment, we designed the GLUE framework for unpaired single-cell multi-omics data integration with superior accuracy and robustness. By modeling regulatory interactions across omics layers explicitly, GLUE uniquely supports integrative regulatory inference for unpaired multi-omics datasets. Notably, in a Bayesian interpretation, the GLUE regulatory inference can be seen as a posterior estimate, which can be continuously refined on the arrival of new data.

Unpaired multi-omics integration shares some conceptual similarities with batch effect correction^50^, but the former is substantially more challenging because of the distinct, omics-specific feature spaces. While feature conversion may seem to be a straightforward solution, the inevitable information loss^19^ can be detrimental. Seurat v3 (ref. ^15^) and bindSC^33^ also devised heuristic strategies to use information in the original feature spaces in addition to converted data, which may explain their improved performance than methods that do not^16,17^. Meanwhile, known cell types have also been used to guide integration via (semi-)supervised learning^51,52^, but this approach incurs substantial limitations in terms of applicability since such supervision is typically unavailable and in many cases serves as the purpose of multi-omics integration per se^29^. Notably, one of these methods was proposed with a similar autoencoder architecture and adversarial alignment^52^, but it relied on matched cell types or clusters to orient the alignment. In fact, GLUE shares more conceptual similarity with coupled matrix factorization methods^20,21^, but with superior performance, which mostly benefits from its deep generative model-based design.

We note that the current framework also works for integrating omics layers with shared features (for example, the integration between scRNA-seq and spatial transcriptomics^53,54^), by using either the same vertex or connected surrogate vertices for shared features in the guidance graph. In addition, cross imputation could also be implemented by chaining encoders and decoders of different omics layers. However, given a recent report that data imputation could induce artifacts and deteriorate the accuracy of gene regulatory inference^55^, such a function may need further investigation.

As a generalizable framework, GLUE features a modular design, where the data and graph autoencoders are independently configurable.

- The data autoencoders in GLUE are customizable with appropriate generative models that conform to omics-specific data distributions. In the current work, we used negative binomial for scRNA-seq and scATAC-seq, and zero-inflated log-normal for snmC-seq (Methods). Nevertheless, generative distributions can be easily reconfigured to accommodate other omics layers, such as protein abundance^56^ and histone modification^57^, and to adopt new advances in data modeling techniques^58^.
- The guidance graphs used in GLUE have currently been limited to multipartite graphs, containing only edges between features of different layers. Nonetheless, graphs, as intuitive and flexible representations of regulatory knowledge, can embody more complex regulatory patterns, including within-modality interactions, nonfeature vertices and multi-relations. Beyond canonical graph convolution, more advanced graph neural network architectures^59–61^ may also be adopted to extract richer information from the regulatory graph. Particularly, recent advances in hypergraph modeling^62,63^ could facilitate the use of prior knowledge on regulatory interactions involving multiple regulators simultaneously, as well as enable regulatory inference for such interactions.

Recent advances in experimental multi-omics technologies have increased the availability of paired data^8–11,34^. While most of the current simultaneous multi-omics protocols still suffer from lower data quality or throughput than that of single-omics methods^64^, paired cells can be highly informative in anchoring different omics layers and should be used in conjunction with unpaired cells whenever available. It is straightforward to extend the GLUE framework to incorporate such pairing information, for example, by adding loss terms that penalize the embedding distances between paired cells^65^. Such an extension may ultimately lead to a solution for the general case of mosaic integration^14^.

Apart from multi-omics integration, we also note that the GLUE framework could be suitable for cross-species integration, especially when distal species are concerned and one-to-one orthologs are limited. Specifically, we may compile all orthologs into a GLUE guidance graph and perform integration without explicit ortholog conversion. Under that setting, the GLUE approach could also be conceptually connected to a recent work called SAMap^66^.

Finally, we note that the inferred regulatory interactions from the current GLUE model are based on the whole input dataset and may be an aggregation of multiple spatiotemporal-specific circuits, especially for data derived from distinct tissues (for example, atlas). Meanwhile, we notice that in parallel to the coarse-scale global model (for example, the whole-atlas integration model), finer-scale regulatory inference could be conducted by training dedicated models on cells from a single tissue, potentially with spatiotemporal-specific prior knowledge incorporated as well^67^. Such a ‘step-wise refinement’ extension would effectively help identify spatiotemporal-specific regulatory circuits and key regulators.

We believe that GLUE, as a modular and generalizable framework, creates an unprecedented opportunity toward effectively delineating gene regulatory maps via large-scale multi-omics integration at single-cell resolution. The whole package of GLUE, along with tutorials and demo cases, is available online at https://github.com/gao-lab/GLUE for the community.

## Methods

### The GLUE framework

We assume that there are *K* different omics layers to be integrated, each with a distinct feature set ${{{\mathcal{V}}}}_k,k = 1,2, \ldots ,K$. For example, in scRNA-seq, ${\mathcal{V}}_k$ is the set of genes, while in scATAC-seq, ${{{\mathcal{V}}}}_k$ is the set of chromatin regions. The data spaces of different omics layers are denoted as ${{{\mathcal{X}}}}_k \subseteq {\Bbb R}^{\left| {{{{\mathcal{V}}}}_k} \right|}$ with varying dimensionalities. We use ${{{\mathbf{x}}}}_k^{(n)} \in {{{\mathcal{X}}}}_k,n = 1,2, \ldots ,N_K$ to denote cells from the *k*th omics layer and ${{{\mathbf{x}}_{k}}_{i}}^{(n)},i \in {{{\mathcal{V}}}}_k$ to denote the observed value of feature *i* of the *k*th layer in the *n*th cell. *N*_*K*_ is the sample size of the *k*th layer. Notably, the cells from different omics layers are unpaired and can have different sample sizes. To avoid cluttering, we drop the superscript (*n*) when referring to an arbitrary cell.

We model the observed data from different omics layers as generated by a low-dimensional latent variable (that is, cell embedding) ${{{\mathbf{u}}}} \in {\Bbb R}^m$:

1 $$\begin{array}{*{20}{c}} {p\left( {{{{\mathbf{x}}}}_k;\theta _k} \right) = {\int} p \left( {{{{\mathbf{x}}}}_k|{{{\mathbf{u}}}};\theta _k} \right)p\left( {{{\mathbf{u}}}} \right){\mathrm{d}}{{{\mathbf{u}}}}} \end{array}$$

where *p*(**u**) is the prior distribution of the latent variable, $p\left( {{{{\mathbf{x}}}}_k|{{{\mathbf{u}}}};\theta _k} \right)$ are learnable generative distributions (that is, data decoders) and *θ*_*k*_ denotes learnable parameters in the decoders. The cell latent variable **u** is shared across different omics layers. In other words, **u** represents the common cell states underlying all omics observations, while the observed data from each layer are generated by a specific type of measurement of the underlying cell states.

With the introduction of variational posteriors $q\left( {{{{\mathbf{u}}}}|{{{\mathbf{x}}}}_k;\phi _k} \right)$ (that is, data encoders, where *ϕ*_*k*_ are learnable parameters in the encoders), model fitting can be efficiently performed by maximizing the following evidence lower bounds:

2 $$\begin{array}{rcl}{{{\mathcal{L}}}}_{{{{\mathcal{X}}}}_k}\left( {\phi _k,\theta _k} \right) & = & {\Bbb E}_{{{{\mathbf{x}}}}_k \sim p_{{{{\mathrm{data}}}}}\left( {{{{\mathbf{x}}}}_k} \right)}\left[ {{\Bbb E}_{{{{\mathbf{u}}}} \sim q\left( {{{{\mathbf{u}}}}|{{{\mathbf{x}}}}_k;\phi _k} \right)}\log p\left( {{{{\mathbf{x}}}}_k|{{{\mathbf{u}}}};\theta _k} \right)}\right. \\ && \left.{ - {{{\mathrm{KL}}}}\left( {q\left( {{{{\mathbf{u}}}}|{{{\mathbf{x}}}}_k;\phi _k} \right)\parallel p\left( {{{\mathbf{u}}}} \right)} \right)} \right]\end{array}$$

Since different autoencoders are independently parameterized and trained on separate data, the cell embeddings learned for different omics layers could have inconsistent semantic meanings unless they are linked properly.

To link the autoencoders, we propose a guidance graph ${{{\mathcal{G}}}} = \left( {{{{\mathcal{V}}}},{{{\mathcal{E}}}}} \right)$, which incorporates prior knowledge about the regulatory interactions among features at distinct omics layers, where ${{{\mathcal{V}}}} = \mathop {\bigcup}\nolimits_{k = 1}^K {{{{\mathcal{V}}}}_k}$ is the universal feature set and ${{{\mathcal{E}}}} = \left\{ {\left( {i,j} \right)|i,j \in {{{\mathcal{V}}}}} \right\}$ is the set of edges. Each edge is also associated with signs and weights, which are denoted as *s*_*ij*_ and *w*_*ij*_, respectively. We require that *w*_*ij*_ ∈ (0,1], which can be interpreted as interaction credibility, and that $s_{ij} \in \left\{ { - 1,1} \right\}$, which specifies the sign of the regulatory interaction. For example, an ATAC peak located near the promoter of a gene is usually assumed to positively regulate its expression, so they can be connected with a positive edge (*s*_*ij*_ = 1). Meanwhile, DNA methylation in the gene promoter is usually assumed to suppress expression, so they can be connected with a negative edge (*s*_*ij*_ = 1). In addition to the connections between features, self-loops are also added for numerical stability, with $s_{ii} = 1,w_{ii} = 1,\forall i \in {{{\mathcal{V}}}}$. The guidance graph is allowed to be a multi-graph, where more than one edge can exist between the same pair of vertices, representing different types of prior regulatory evidence.

We treat the guidance graph as observed variable and model it as generated by low-dimensional feature latent variables (that is, feature embeddings) ${{{\mathbf{v}}}}_i \in {\Bbb R}^m,i \in {{{\mathcal{V}}}}$. Furthermore, differing from the previous model, we now model **x**_*k*_ as generated by the combination of feature latent variables ${{{\mathbf{v}}}}_i \in {\Bbb R}^m,i \in {{{\mathcal{V}}}}_k$ and the cell latent variable ${{{\mathbf{u}}}} \in {\Bbb R}^m$. For convenience, we introduce the notation ${{{\mathbf{V}}}} \in {\Bbb R}^{m \times \left| {{{\mathcal{V}}}} \right|}$, which combines all feature embeddings into a single matrix. The model likelihood can thus be written as:

3 $$\begin{array}{*{20}{c}} {p\left( {{{{\mathbf{x}}}}_k,{{{\mathcal{G}}}};\theta _k,\theta _{{{\mathcal{G}}}}} \right) = {\int} p \left( {{{{\mathbf{x}}}}_k|{{{\mathbf{u}}}},{{{\mathbf{V}}}};\theta _k} \right)p\left( {{{{\mathcal{G}}}}|{{{\mathbf{V}}}};\theta _{{{\mathcal{G}}}}} \right)p\left( {{{\mathbf{u}}}} \right)p\left( {{{\mathbf{V}}}} \right){\mathrm{d}}{{{\mathbf{u}}}}{\mathrm{d}}{{{\mathbf{V}}}}} \end{array}$$

where $p\left( {{{{\mathbf{x}}}}_k|{{{\mathbf{u}}}},{{{\mathbf{V}}}};\theta _k} \right)$ and $p\left( {{{{\mathcal{G}}}}|{{{\mathbf{V}}}};\theta _{{{\mathcal{G}}}}} \right)$ are learnable generative distributions for the omics data (that is, data decoders) and knowledge graph (that is, graph decoder), respectively. *θ*_*k*_ and $\theta _{{{\mathcal{G}}}}$ are learnable parameters in the decoders. *p*(**u**) and *p*(**V**) are the prior distributions of the cell latent variable and feature latent variables, respectively, which are fixed as standard normal distributions for simplicity:

4 $$\begin{array}{*{20}{c}} {p\left( {{{\mathbf{u}}}} \right) = N\left( {{{{\mathbf{u}}}};\mathbf{0},{{{\mathbf{I}}}}_m} \right)} \end{array}$$

5 $$\begin{array}{*{20}{c}} {p\left( {{{{\mathbf{v}}}}_i} \right) = N\left( {{{{\mathbf{v}}}}_i;\mathbf{0},{{{\mathbf{I}}}}_m} \right),p\left( {{{\mathbf{V}}}} \right) = \mathop {\prod }\limits_{i \in {{{\mathcal{V}}}}} p\left( {{{{\mathbf{v}}}}_i} \right)} \end{array}$$

although alternatives may also be used^68^. For convenience, we also introduce the notation ${{{\mathbf{V}}}}_k \in {\Bbb R}^{m \times \left| {{{{\mathcal{V}}}}_k} \right|}$, which contains only feature embeddings in the *k*th omics layer, and **u**_*k*_, which emphasizes that the cell embedding is from a cell in the *k*th omics layer.

The graph likelihood $p\left( {{{{\mathcal{G}}}}|{{{\mathbf{V}}}};\theta _{{{\mathcal{G}}}}} \right)$ (that is, graph decoder) is defined as:

6 $$\begin{array}{rcl} {\log p\left( {{{{\mathcal{G}}}}|{{{\mathbf{V}}}};\theta _{{{\mathcal{G}}}}} \right)} = {{\Bbb E}_{i,j \sim p\left( {i,j;w_{ij}} \right)}} \\ {\left[ {\log \sigma \left( {s_{ij} {{{\mathbf{v}}}}_i^ \top {{{\mathbf{v}}}}_j} \right) + {\Bbb E}_{j\prime \sim p_{{{{\mathrm{ns}}}}}\left( {j\prime |i} \right)}\log \left( {1 - \sigma \left( {s_{ij} {{{\mathbf{v}}}}_i^ \top {{{\mathbf{v}}}}_{j\prime }} \right)} \right)} \right]} \end{array}$$

where *σ* is the sigmoid function and *p*_ns_ is a negative sampling distribution^69^. Here the graph likelihood has no trainable parameters, so $\theta _{{{\mathcal{G}}}} = \emptyset$. In other words, we first sample the edges (*i*, *j*) with probabilities proportional to the edge weights and then sample vertices *j*′ that are not connected to *i* and treat them as if $s_{ij\prime } = s_{ij}$. When maximizing the graph likelihood, the inner products between features are maximized or minimized (per edge sign) based on the Bernoulli distribution. For example, ATAC peaks located near the promoter of a gene would be encouraged to have similar embeddings to that of the gene, while DNA methylation in the gene promoter would be encouraged to have a dissimilar embedding to that of the gene.

The data likelihoods $p\left( {{{{\mathbf{x}}}}_k|{{{\mathbf{u}}}},{{{\mathbf{V}}}};\theta _k} \right)$ (that is, data decoders) in equation (3) are built on the inner product between the cell embedding **u** and feature embeddings **V**_*k*_. Thus, analogous to the loading matrix in principal component analysis (PCA), the feature embeddings **V**_*k*_ confer semantic meanings for the cell embedding space. As **V**_*k*_ are modulated by interactions among omics features in the guidance graph, the semantic meanings become linked. While this linearity limits decoder capacity, our empirical evaluations show that it is well compensated by the nonlinear encoders, producing high-quality multi-omics alignments (Fig. 2, Extended Data Figs. 1–4 and Supplementary Figs. 1–7). The exact formulation of data likelihood depends on the omics data distribution. For example, for count-based scRNA-seq and scATAC-seq data, we used the negative binomial (NB) distribution:

7 $$\begin{array}{*{20}{c}} {p\left( {{{{\mathbf{x}}}}_k|{{{\mathbf{u}}}},{{{\mathbf{V}}}};\theta _k} \right) = \mathop {\prod }\limits_{i \in {{{\mathcal{V}}}}_k} {{{\mathrm{NB}}}}\left( {{{\mathbf{x}}_{k}}_{i};\mathbf{\mu} _i,\mathbf{\theta} _i} \right)} \end{array}$$

8 $$\begin{array}{*{20}{c}} {{\mathrm{NB}}\left( {{{\mathbf{x}}_{k}}_{i};{{{\mathbf{\mu }}}}_i,{{{\mathbf{\theta }}}}_i} \right) = \frac{{{{{\mathrm{{\Gamma}}}}}\left( {{{\mathbf{x}}_{k}}_{i} + {{{\mathbf{\theta }}}}_i} \right)}}{{{{{\mathrm{{\Gamma}}}}}\left( {{{{\mathbf{\theta }}}}_i} \right){{{\mathrm{{\Gamma}}}}}\left( {{{\mathbf{x}}_{k}}_{i} + 1} \right)}}\left( {\frac{{{{{\mathbf{\mu }}}}_i}}{{{{{\mathbf{\theta }}}}_i + {{{\mathbf{\mu }}}}_i}}} \right)^{{\mathbf{x}_{k}}_{i}}\left( {\frac{{{{{\mathbf{\theta }}}}_i}}{{{{{\mathbf{\theta }}}}_i + {{{\mathbf{\mu }}}}_i}}} \right)^{{{{\mathbf{\theta }}}}_i}} \end{array}$$

9 $$\begin{array}{*{20}{c}} {{{{\mathbf{\mu }}}}_i = {{{\mathrm{Softmax}}}}_i\left( {{{{\mathbf{\alpha }}}} \odot {{{\mathbf{V}}}}_k^ \top {{{\mathbf{u}}}} + {{{\mathbf{\beta }}}}} \right) \cdot \mathop {\sum }\limits_{j \in {{{\mathcal{V}}}}_k} {\mathbf{x}_{k}}_{j}} \end{array}$$

where ${{{\mathbf{\mu }}}},{{{\mathbf{\theta }}}} \in {\Bbb R}_ + ^{\left| {{{{\mathcal{V}}}}_k} \right|}$ are the mean and dispersion of the negative binomial distribution, respectively, ${{{\mathbf{\alpha }}}} \in {\Bbb R}_ + ^{\left| {{{{\mathcal{V}}}}_k} \right|},{{{\mathbf{\beta }}}} \in {\Bbb R}^{\left| {{{{\mathcal{V}}}}_k} \right|}$ are scaling and bias factors, ⊙ is the Hadamard product, Softmax_*i*_ represents the *i*th dimension of the softmax output and $\mathop {\sum}\nolimits_{j \in {{{\mathcal{V}}}}_k} {{\mathbf{x}_{k}}_{j}}$ gives the total count in the cell. Taking softmax and then multiplying by total count ensures that the library size of reconstructed data matches the original^30^. The set of learnable parameters is $\theta _k = \left\{ {{{{\mathbf{\theta }}}},{{{\mathbf{\alpha }}}},{{{\mathbf{\beta }}}}} \right\}$. Analogously, many other distributions can also be supported, as long as we can parameterize the means of the distributions by feature-cell inner products.

For efficient inference and optimization, we introduce the following factorized variational posterior:

10 $$\begin{array}{*{20}{c}} {q\left( {{{{\mathbf{u}}}},{{{\mathbf{V}}}}|{{{\mathbf{x}}}}_k,{{{\mathcal{G}}}};\phi _k,\phi _{{{\mathcal{G}}}}} \right) = q\left( {{{{\mathbf{u}}}}|{{{\mathbf{x}}}}_k;\phi _k} \right) \cdot q\left( {{{{\mathbf{V}}}}|{{{\mathcal{G}}}};\phi _{{{\mathcal{G}}}}} \right)} \end{array}$$

The graph variational posterior $q\left( {{{{\mathbf{V}}}}|{{{\mathcal{G}}}};\phi _{{{\mathcal{G}}}}} \right)$ (that is, graph encoder) is modeled as diagonal-covariance normal distributions parameterized by a graph convolutional network^70^:

11 $$\begin{array}{*{20}{c}} {q\left( {{{{\mathbf{V}}}}|{{{\mathcal{G}}}};\phi _{{{\mathcal{G}}}}} \right) = \mathop {\prod }\limits_{i \in {{{\mathcal{V}}}}} q\left( {{{{\mathbf{v}}}}_i|{{{\mathcal{G}}}};\phi _{{{\mathcal{G}}}}} \right)} \end{array}$$

12 $$\begin{array}{*{20}{c}} {q\left( {{{{\mathbf{v}}}}_i|{{{\mathcal{G}}}};\phi _{{{\mathcal{G}}}}} \right) = N\left( {{{{\mathbf{v}}}}_i;{{{\mathrm{GCN}}}}_{{{{\mathbf{\mu }}}}_i}\left( {{{{\mathcal{G}}}};\phi _{{{\mathcal{G}}}}} \right),{{{\mathrm{GCN}}}}_{{{{\mathbf{\sigma }}}}_i^2}\left( {{{{\mathcal{G}}}};\phi _{{{\mathcal{G}}}}} \right)} \right)} \end{array}$$

where $\phi _{{{\mathcal{G}}}}$ represents the learnable parameters in the graph convolutional network (GCN) encoder.

The variational data posteriors $q\left( {{{{\mathbf{u}}}}|{{{\mathbf{x}}}}_k;\phi _k} \right)$ (that is, data encoders) are modeled as diagonal-covariance normal distributions parameterized by multilayer perceptron (MLP) neural networks:

13 $$\begin{array}{*{20}{c}} {q\left( {{{{\mathbf{u}}}}|{{{\mathbf{x}}}}_k,{{{\mathbf{V}}}}_k;\phi _k} \right) = N\left( {{{{\mathbf{u}}}};{{{\mathrm{MLP}}}}_{k,{{{\mathbf{\mu }}}}}\left( {{{{\mathbf{x}}}}_k;\phi _k} \right),{{{\mathrm{MLP}}}}_{k,{{{\mathbf{\upsigma }}}}^2}\left( {{{{\mathbf{x}}}}_k;\phi _k} \right)} \right)} \end{array}$$

where *ϕ*_*k*_ is the set of learnable parameters in the multilayer perceptron encoder of the *k*th omics layer.

Model fitting can then be performed by maximizing the following evidence lower bound:

14 $$\begin{array}{*{20}{c}} {\mathop {\sum}\limits_{k = 1}^K {{\Bbb E}_{{{{\mathbf{x}}}}_k \sim p_{{{{\mathrm{data}}}}}\left( {{{{\mathbf{x}}}}_k} \right)}} \left[ {\begin{array}{*{20}{c}} {{\Bbb E}_{{{{\mathbf{u}}}} \sim q\left( {{{{\mathbf{u}}}}|{{{\mathbf{x}}}}_k;\phi _k} \right),{{{\mathbf{V}}}} \sim q\left( {{{{\mathbf{V}}}}|{{{\mathcal{G}}}};\phi _{{{\mathcal{G}}}}} \right)}\log p\left( {{{{\mathbf{x}}}}_k|{{{\mathbf{u}}}},{{{\mathbf{V}}}};\theta _k} \right)p\left( {{{{\mathcal{G}}}}|{{{\mathbf{V}}}};\theta _{{{\mathcal{G}}}}} \right)} \\ { - \mathrm{KL}\left( {q\left( {{{{\mathbf{u}}}}|{{{\mathbf{x}}}}_k;\phi _k} \right)q\left( {{{{\mathbf{V}}}}|{{{\mathcal{G}}}};\phi _{{{\mathcal{G}}}}} \right)\parallel p\left( {{{\mathbf{u}}}} \right)p\left( {{{\mathbf{V}}}} \right)} \right)} \end{array}} \right]} \end{array}$$

which can be further rearranged into the following form:

15 $$\begin{array}{*{20}{c}} {K \cdot {{{\mathcal{L}}}}_{{{\mathcal{G}}}}\left( {\theta _{{{\mathcal{G}}}},\phi _{{{\mathcal{G}}}}} \right) + \mathop {\sum}\limits_{k = 1}^K {{{{\mathcal{L}}}}_{{{{\mathcal{X}}}}_k}} \left( {\theta _k,\phi _k,\phi _{{{\mathcal{G}}}}} \right)} \end{array}$$

where we have

16 $$\begin{array}{rcl}{{{\mathcal{L}}}}_{{{{\mathcal{X}}}}_k}\left( {\theta _k,\phi _k,\phi _{{{\mathcal{G}}}}} \right) = {\Bbb E}_{{{{\mathbf{x}}}}_k \sim p_{{{{\mathrm{data}}}}}\left( {{{{\mathbf{x}}}}_k} \right)} \\ \left[ {{\Bbb E}_{{{{\mathbf{u}}}} \sim q\left( {{{{\mathbf{u}}}}|{{{\mathbf{x}}}}_k;\phi _k} \right),{{{\mathbf{V}}}} \sim q\left( {{{{\mathbf{V}}}}|{{{\mathcal{G}}}};\phi _{{{\mathcal{G}}}}} \right)}\log p\left( {{{{\mathbf{x}}}}_k|{{{\mathbf{u}}}},{{{\mathbf{V}}}};\theta _k} \right) - {{{\mathrm{KL}}}}\left( {q\left( {{{{\mathbf{u}}}}|{{{\mathbf{x}}}}_k;\phi _k} \right)\parallel p\left( {{{\mathbf{u}}}} \right)} \right)} \right]\end{array}$$

17 $$\begin{array}{*{20}{c}} {{{{\mathcal{L}}}}_{{{\mathcal{G}}}}\left( {\theta _{{{\mathcal{G}}}},\phi _{{{\mathcal{G}}}}} \right) = {\Bbb E}_{{{{\mathbf{V}}}} \sim q\left( {{{{\mathbf{V}}}}|{{{\mathcal{G}}}};\phi _{{{\mathcal{G}}}}} \right)}\log p\left( {{{{\mathcal{G}}}}|{{{\mathbf{V}}}};\theta _{{{\mathcal{G}}}}} \right) - \mathrm{KL}\left( {q\left( {{{{\mathbf{V}}}}|{{{\mathcal{G}}}};\phi _{{{\mathcal{G}}}}} \right)\parallel p\left( {{{\mathbf{V}}}} \right)} \right)} \end{array}$$

Below, for convenience, we denote the union of all encoder parameters as $\phi = \left( {\mathop {\bigcup}\nolimits_{k = 1}^K {\phi _k} } \right) \cup \phi _{{{\mathcal{G}}}}$ and the union of all decoder parameters as $\theta = \left( {\mathop {\bigcup}\nolimits_{k = 1}^K {\theta _k} } \right) \cup \theta _{{{\mathcal{G}}}}$.

To ensure the proper alignment of different omics layers, we use the adversarial alignment strategy^31,71^. A discriminator D with a *K*-dimensional softmax output is introduced, which predicts the omics layers of cells based on their embeddings **u**. The discriminator D is trained by minimizing the multiclass classification cross entropy:

18 $$\begin{array}{*{20}{c}} {{{{\mathcal{L}}}}_{{{\mathrm{D}}}}\left( {\phi ,\psi } \right) = - \frac{1}{K}\mathop {\sum }\limits_{k = 1}^K {\Bbb E}_{{{{\mathbf{x}}}}_k \sim p_{{{{\mathrm{data}}}}}\left( {{{{\mathbf{x}}}}_k} \right)}{\Bbb E}_{{{{\mathbf{u}}}} \sim q\left( {{{{\mathbf{u}}}}|{{{\mathbf{x}}}}_k;\phi _k} \right)}\log {{{\mathrm{D}}}}_k\left( {{{{\mathbf{u}}}};\psi } \right)} \end{array}$$

where D_*k*_ represents the *k*th dimension of the discriminator output and *ψ* is the set of learnable parameters in the discriminator. The data encoders can then be trained in the opposite direction to fool the discriminator, ultimately leading to the alignment of cell embeddings from different omics layers^72^.

The overall training objective of GLUE thus consists of:

19 $$\begin{array}{*{20}{c}} {\mathop {{\min }}\limits_\psi \lambda _{{{\mathrm{D}}}} \cdot {{{\mathcal{L}}}}_{{{\mathrm{D}}}}\left( {\phi ,\psi } \right)} \end{array}$$

20 $$\begin{array}{*{20}{c}} {\mathop {{\max }}\limits_{\theta ,\phi } \lambda _{{{\mathrm{D}}}} \cdot {{{\mathcal{L}}}}_{{{\mathrm{D}}}}\left( {\phi ,\psi } \right) + \lambda _{{{\mathcal{G}}}}K \cdot {{{\mathcal{L}}}}_{{{\mathcal{G}}}}\left( {\theta _{{{\mathcal{G}}}},\phi _{{{\mathcal{G}}}}} \right) + \mathop {\sum }\limits_{k = 1}^K {{{\mathcal{L}}}}_{{{{\mathcal{X}}}}_k}\left( {\theta _k,\phi _k,\phi _{{{\mathcal{G}}}}} \right)} \end{array}$$

The two hyperparameters *λ*_D_ and $\lambda _{{{\mathcal{G}}}}$ control the contributions of adversarial alignment and graph-based feature embedding, respectively. We use stochastic gradient descent to train the GLUE model. Each stochastic gradient descent iteration is divided into two steps. In the first step, the discriminator is updated according to objective equation (19). In the second step, the data and graph autoencoders are updated according to equation (20). The RMSprop optimizer with no momentum term is used to ensure the stability of adversarial training.

### Weighted adversarial alignment

As shown in previous work^31^, canonical adversarial alignment amounts to minimizing a generalized form of Jensen–Shannon divergence among the cell embedding distributions of different omics layers:

21 $$\frac{1}{K}\mathop {\sum}\limits_{k = 1}^K {{{{\mathrm{KL}}}}} \left( {q_k({{{\mathbf{u}}}})||\frac{1}{K}\mathop {\sum}\limits_{k = 1}^K {q_k} ({{{\mathbf{u}}}})} \right)$$

where $q_k\left( {{{\mathbf{u}}}} \right) = {\Bbb E}_{{{{\mathbf{x}}}}_k \sim p_{{{{\mathrm{data}}}}}\left( {{{{\mathbf{x}}}}_k} \right)}q\left( {{{{\mathbf{u}}}}|{{{\mathbf{x}}}}_k;\phi _k} \right)$ represents the marginal cell embedding distribution of the *k*th layer. Without other loss terms, equation (21) converges at perfect alignment, that is, when $q_i\left( {{{\mathbf{u}}}} \right) = q_j\left( {{{\mathbf{u}}}} \right),\forall i \ne j$. This can be problematic when cell type compositions differ dramatically across different layers, for example, in the cell atlas integration. To address this issue, we added cell-specific weights *w*^(*n*)^ to the discriminator loss in equation (18):

22 $$\begin{array}{*{20}{c}} {{{{\mathcal{L}}}}_{{{\mathrm{D}}}}\left( {\phi ,\psi } \right) = - \frac{1}{K}\mathop {\sum }\limits_{k = 1}^K \frac{1}{{W_k}}\mathop {\sum }\limits_{n = 1}^{N_k} w^{\left( n \right)} \cdot {\Bbb E}_{{{{\mathbf{u}}}} \sim q\left( {{{{\mathbf{u}}}}|{{{\mathbf{x}}}}_k^{\left( n \right)};\phi _k} \right)}\log {{{\mathrm{D}}}}_k\left( {{{{\mathbf{u}}}};\psi } \right)} \end{array}$$

where the normalizer $W_k = \mathop {\sum}\nolimits_{n = 1}^{N_k} {w^{\left( n \right)}}$. The adversarial alignment still amounts to minimizing equation (21) but with weighted marginal cell embedding distributions $q_k\left( {{{\mathbf{u}}}} \right) = \frac{1}{{W_k}}\mathop {\sum}\limits_{n = 1}^{N_k} {w^{\left( n \right)}} q\left( {{{{\mathbf{u}}}}|{{{\mathbf{x}}}}_k^{\left( n \right)};\phi _k} \right)$. By assigning appropriate weights to balance the cell distributions across different layers, the optimum of $q_i\left( {{{\mathbf{u}}}} \right) = q_j\left( {{{\mathbf{u}}}} \right),\forall i \ne j$ could be much closer to the desired alignment.

To obtain the balancing weights in an unsupervised manner, we devised the following two-stage training procedure. First, we pretrain the GLUE model with constant weight $w^{\left( n \right)} = 1$, during which noise ${\boldsymbol{\epsilon}} \sim {{{\mathcal{N}}}}\left( {{\boldsymbol{\epsilon}} ;\mathbf{0},{\mathbf{\Sigma}}} \right)$ was added to the cell embeddings before passing to the discriminator. We set **∑** to be 1.5× the empirical variance of cell embeddings in each minibatch, which helps produce a coarse alignment immune to composition imbalance. Then, we cluster the coarsely aligned cell embeddings per omics layer using Leiden clustering. The balancing weight *w*_*i*_ for cells in cluster *i* is computed as:

23 $$\begin{array}{*{20}{c}} {w_i = \frac{{\mathop {\sum }\nolimits_{k_i \ne k_j} f\left( {{{{\mathbf{u}}}}_i,{{{\mathbf{u}}}}_j} \right)}}{{n_i}}} \end{array}$$

24 $$\begin{array}{*{20}{c}} {f\left( {{{{\mathbf{u}}}}_i,{{{\mathbf{u}}}}_j} \right) = \left\{ {\begin{array}{*{20}{l}} {\cos \left( {{{{\mathbf{u}}}}_i,{{{\mathbf{u}}}}_j} \right)^4,} \hfill & {{\mathrm{cos}}({{{\mathbf{u}}}}_i,{{{\mathbf{u}}}}_j) > 0.5} \hfill \\ {0,} \hfill & {{\mathrm{otherwise}}} \hfill \end{array}} \right.} \end{array}$$

where **u**_*i*_ is the average cell embedding of cluster *i*, *k*_*i*_ denotes the omics layer of cluster *i*, and *n*_*i*_ is the number of cells in cluster *i*. In other words, we sum up the cosine similarities (raised to the power of 4 to increase contrast) between cluster *i* and all its matching clusters in other layers with cosine similarity >0.5, and then normalize by cluster size, which effectively balances the contribution of matching clusters regardless of their sizes. In the second stage, we fine-tune the GLUE model with the estimated balancing weights, during which the additive noise ${\boldsymbol{\epsilon}} \sim {{{\mathcal{N}}}}\left( {{\boldsymbol{\epsilon}} ;\mathbf{0},\tau \cdot {\mathbf{\Sigma}}} \right)$ gradually anneals to 0 (with *τ* starting at 1 and decreasing linearly per epoch until 0). The number of annealing epochs was set automatically based on the data size and learning rate to match a learning progress equivalent to 4,000 iterations at a learning rate of 0.002.

All benchmarks and case studies in the study were conducted with the two-stage training procedure as described above, regardless of whether the dataset being used is balanced or not.

### Batch effect correction

To handle batch effect within omics layers, we incorporate batch as a covariate of the data decoders. Assuming $b \in \left\{ {1,2, \ldots ,B} \right\}$, is the batch index, where *B* is the total number of batches, the decoder likelihood is extended to $p\left( {{{{\mathbf{x}}}}_k|{{{\mathbf{u}}}},{{{\mathbf{V}}}},b;\theta _k} \right)$. Specifically, this is achieved by converting learnable parameters in the data decoder to be batch-dependent. For example, in the case of a negative binomial decoder, the network now uses batch-specific **α**, **β** and **θ** parameters:

25 $$\begin{array}{*{20}{c}} {p\left( {{{{\mathbf{x}}}}_k|{{{\mathbf{u}}}},{{{\mathbf{V}}}},b;\theta _k} \right) = \mathop {\prod }\limits_{i \in {{{\mathcal{V}}}}_k} {{{\mathrm{NB}}}}\left( {{\mathbf{x}_{k}}_{i};{{{\mathbf{\mu }}}}_i,{\mathbf{\theta }_{b}}_{i}} \right)} \end{array}$$

26 $$\begin{array}{*{20}{c}} {{\mathrm{NB}}\left( {{\mathbf{x}_{k}}_{i};{{{\mathbf{\mu }}}}_i,{\mathbf{\theta }_{b}}_{i}} \right) = \frac{{{\Gamma}\left( {{\mathbf{x}_{k}}_{i} + {\mathbf{\theta }_{b}}_{i}} \right)}}{{{\Gamma}\left( {{\mathbf{\theta }_{b}}_{i}} \right){\Gamma}\left( {{\mathbf{x}_{k}}_{i} + 1} \right)}}\left( {\frac{{{{{\mathbf{\mu }}}}_i}}{{{\mathbf{\theta }_{b}}_{i} + {{{\mathbf{\mu }}}}_i}}} \right)^{{\mathbf{x}_{k}}_{i}}\left( {\frac{{{\mathbf{\theta }_{b}}_{i}}}{{{\mathbf{\theta }_{b}}_{i} + {{{\mathbf{\mu }}}}_i}}} \right)^{{\mathbf{\theta }_{b}}_{i}}} \end{array}$$

27 $$\begin{array}{*{20}{c}} {{{{\mathbf{\mu }}}}_i = {{{\mathrm{Softmax}}}}_i\left( {{{{\mathbf{\alpha }}}}_b \odot {{{\mathbf{V}}}}_k^ \top {{{\mathbf{u}}}} + {{{\mathbf{\beta }}}}_b} \right) \cdot \mathop {\sum }\limits_{j \in {{{\mathcal{V}}}}_k} {\mathbf{x}_{k}}_{j}} \end{array}$$

where ${{{\mathbf{\alpha }}}} \in {\Bbb R}_ + ^{B \times \left| {{{{\mathcal{V}}}}_k} \right|},{{{\mathbf{\beta }}}} \in {\Bbb R}^{B \times \left| {{{{\mathcal{V}}}}_k} \right|},{{{\mathbf{\theta }}}} \in {\Bbb R}_ + ^{B \times \left| {{{{\mathcal{V}}}}_k} \right|}$, and **α**_*b*_, **β**_*b*_, **θ**_*b*_ are the *b*th row of **α**, **β**, **θ**. Other probabilistic decoders can also be extended in similar ways.

### Implementation details

We applied linear dimensionality reduction using canonical methods such as PCA (for scRNA-seq) or LSI (latent semantic indexing, for scATAC-seq) as the first transformation layers of the data encoders (note that the decoders were still fitted in the original feature spaces). This effectively reduced model size and enabled a modular input, so advanced dimensionality reduction or batch effect correction methods can also be used instead as preprocessing steps for GLUE integration.

During model training, 10% of the cells were used as the validation set. In the final stage of training, the learning rate would be reduced by factors of 10 if the validation loss did not improve for consecutive epochs. Training would be terminated if the validation loss still did not improve for consecutive epochs. The patience for learning rate reduction, training termination and the maximal number of training epochs were automatically set based on the data size and learning rate to match a learning progress equivalent to 1,000, 2,000 and 16,000 iterations at a learning rate of 0.002, respectively.

For all benchmarks and case studies with GLUE, we used the default hyperparameters unless explicitly stated. The set of default hyperparameters is presented in Extended Data Fig. 3.

### Integration consistency score

The integration consistency score is a measure of consistency between the integrated multi-omics data and the guidance graph. First, we jointly cluster cells from all omics layers in the aligned cell embedding space using *k*-means. For each omics layer, the cells in each cluster are aggregated into a metacell. The metacells are established as paired samples, based on which feature correlation can be computed. Using the paired metacells, we then compute the Spearman’s correlation for each edge in the guidance graph. The integration consistency score is defined as the average correlation across all graph edges, negated per edge sign and weighted by edge weight.

### Systematic benchmarks

UnionCom^23^, Pamona^24^ and GLUE were executed using the Python packages ‘unioncom’ (v.0.3.0), ‘Pamona’ (v.0.1.0) and ‘scglue’ (v.0.2.0), respectively. MMD-MA^25^ was executed using the Python script provided at https://bitbucket.org/noblelab/2020_mmdma_pytorch. Online iNMF^16^, LIGER^17^, Harmony^18^, bindSC^33^, and Seurat v3 (ref. ^15^) were executed using the R packages ‘rliger’ (v.1.0.0), ‘rliger’ (v.1.0.0), ‘harmony’ (v.0.1.0), ‘bindSC’ (v.1.0.0) and ‘Seurat’ (v.4.0.2), respectively. For each method, we used the default hyperparameter settings and data preprocessing steps as recommended. For the scRNA-seq data, 2,000 highly variable genes were selected using the Seurat ‘vst’ method. We used two separate schemes to construct the guidance graph. In the standard scheme, we connected ATAC peaks with RNA genes via positive edges if they overlapped in either the gene body or proximal promoter regions (defined as 2 kb upstream from the TSS). In an alternative scheme involving larger genomic windows, we connected ATAC peaks with RNA genes via positive edges if the peaks are within 150 kb of the proximal gene promoters; the edges were weighted by a power-law function $w = \left( {d + 1} \right)^{ - 0.75}$ (*d* is the genomic distance in kb), which has been proposed to model the probability of chromatin contact^42,43^. For the methods that require feature conversion (online iNMF, LIGER, bindSC and Seurat v.3), we converted the scATAC-seq data to gene-level activity scores by summing up counts in the ATAC peaks connected to specific genes in the guidance graph. Notably, online iNMF and LIGER also recommend an alternative way of ATAC feature conversion, that is, directly counting ATAC fragments falling in gene body and promoter regions without resorting to ATAC peaks (https://htmlpreview.github.io/?https://github.com/welch-lab/liger/blob/master/vignettes/Integrating_scRNA_and_scATAC_data.html), which we abbreviate to FiG (fragments in genes). We also tested the FiG feature conversion method with online iNMF and LIGER whenever applicable.

Mean average precision (MAP) was used to evaluate the cell type resolution. Supposing that the cell type of the *i*th cell is *y*^(*i*)^ and that the cell types of its *K* ordered nearest neighbors are $y_1^{\left( i \right)},y_2^{\left( i \right)}, \ldots, y_K^{\left( i \right)}$, the mean average precision is then defined as follows:

28 $$\begin{array}{*{20}{c}} {{\mathrm{MAP}} = \frac{1}{N}\mathop {\sum}\limits_{i = 1}^N {{{{\mathrm{AP}}}}^{\left( i \right)}} } \end{array}$$

29 $$\begin{array}{*{20}{c}} {{{{\mathrm{AP}}}}^{\left( i \right)} = \left\{ {\begin{array}{*{20}{l}} {\frac{{\mathop {\sum }\nolimits_{k = 1}^K 1_{y^{\left( i \right)} = y_k} \cdot \frac{{\mathop {\sum }\nolimits_{j = 1}^k 1_{y^{\left( i \right)} = y_j^{\left( i \right)}}}}{k}}}{{\mathop {\sum }\nolimits_{k = 1}^K 1_{y^{\left( i \right)} = y_k^{\left( i \right)}}}},} \hfill & {{\mathrm{if}}\,\mathop {\sum }\limits_{k = 1}^K 1_{y^{\left( i \right)} = y_k^{\left( i \right)}} > 0} \hfill \\ {0,} \hfill & {{\mathrm{otherwise}}} \hfill \end{array}} \right.} \end{array}$$

where $1_{y^{\left( i \right)} = y_k^{\left( i \right)}}$ is an indicator function that equals 1 if $y^{\left( i \right)} = y_k^{\left( i \right)}$ and 0 otherwise. For each cell, average precision (AP) computes the average cell type precision up to each cell type-matched neighbor, and mean average precision is the average average precision across all cells. We set *K* to 1% of the total number of cells in each dataset. Mean average precision has a range of 0 to 1, and higher values indicate better cell type resolution.

Cell type ASW (average silhouette width) was also used to evaluate the cell type resolution, which was defined as in a recent benchmark study^73^:

30 $$\begin{array}{*{20}{c}} {{\mathrm{cell}}\,{\mathrm{type}}\,{\mathrm{ASW}} = \frac{1}{2}\left( {\frac{{\mathop {\sum }\nolimits_{i = 1}^N s_{{{{\mathrm{cell}}}}\,{{{\mathrm{type}}}}}^{\left( i \right)}}}{N} + 1} \right)} \end{array}$$

where $s_{{{{\mathrm{cell}}}}\,{{{\mathrm{type}}}}}^{\left( i \right)}$ is the cell type silhouette width for the *i*th cell, and *N* is the total number of cells. Cell type ASW has a range of 0 to 1, and higher values indicate better cell type resolution.

Neighbor consistency (NC) was used to evaluate the preservation of single-omics data variation after multi-omics integration and was defined following a previous study^74^:

31 $$\begin{array}{*{20}{c}} {{\mathrm{NC}} = \frac{1}{N}\mathop {\sum }\limits_{i = 1}^N \frac{{\left| {{{{\mathrm{NNS}}}}^{\left( {{{\mathrm{i}}}} \right)} \cap {{{\mathrm{NNI}}}}^{\left( {{{\mathrm{i}}}} \right)}} \right|}}{{\left| {{{{\mathrm{NNS}}}}^{\left( {{{\mathrm{i}}}} \right)} \cup {{{\mathrm{NNI}}}}^{\left( {{{\mathrm{i}}}} \right)}} \right|}}} \end{array}$$

where NNS^(i)^ is the set of k-nearest neighbors for cell *i* in the single-omics data, NNI^(i)^ is the set of *K*-nearest neighbors for the *i*th cell in the integrated space, and *N* is the total number of cells. We set *K* to 1% of the total number of cells in each dataset. Neighbor consistency has a range of 0 to 1, and higher values indicate better preservation of data variation.

#### Biology conservation

Mean average precision, cell type ASW and neighbor consistency all measure biology conservation of the data integration. Following the procedure from the recent benchmark study^73^, we first conduct min-max scaling for each of the metrics and then compute the average across the three to summarize them into a single metric representing biology conservation:

32 $$\begin{array}{*{20}{c}} {{\mathrm{biology}}\,{\mathrm{conservation}} = \frac{{{{{\mathrm{scale}}}}\left( {{{{\mathrm{MAP}}}}} \right) + {{{\mathrm{scale}}}}\left( {{{{\mathrm{cell}}}}\,{{{\mathrm{type}}}}\,{{{\mathrm{ASW}}}}} \right) + {{{\mathrm{scale}}}}\left( {{{{\mathrm{NC}}}}} \right)}}{3}} \end{array}$$

Seurat alignment score (SAS) was used to evaluate the extent of mixing among omics layers and was computed as described in the original paper^75^:

33 $$\begin{array}{*{20}{c}} {{\mathrm{SAS}} = 1 - \frac{{\bar x - \frac{K}{N}}}{{K - \frac{K}{N}}}} \end{array}$$

where $\bar x$ is the average number of cells from the same omics layer among the *K*-nearest neighbors (different layers were first subsampled to the same number of cells as the smallest layer), and *N* is the number of omics layers. We set *K* to 1% of the subsampled cell number. Seurat alignment score has a range of 0 to 1, and higher values indicate better mixing.

Omics layer ASW was also used to evaluate the extend of mixing among omics layers and was defined as in a recent benchmark study^73^:

34 $$\begin{array}{*{20}{c}} {{\mathrm{omics}}\,{\mathrm{layer}}\,{\mathrm{ASW}} = \frac{1}{M}\mathop {\sum}\limits_{j = 1}^M {{{{\mathrm{omics}}}}\,{{{\mathrm{layer}}}}\,{{{\mathrm{ASW}}}}_{{{\mathrm{j}}}}} } \end{array}$$

35 $$\begin{array}{*{20}{c}} {{{{\mathrm{omics}}}}\,{{{\mathrm{layer}}}}\,{{{\mathrm{ASW}}}}_{{{\mathrm{j}}}} = \frac{1}{{N_j}}\mathop {\sum }\limits_{i = 1}^{N_j} 1 - \left| {s_{{{{\mathrm{omics}}}}\,{{{\mathrm{layer}}}}}^{\left( i \right)}} \right|} \end{array}$$

where $s_{{{{\mathrm{omics}}}}\,{{{\mathrm{layer}}}}}^{\left( i \right)}$ is the omics layer silhouette width for the *i*th cell, *N*_*j*_ is the number of cells in cell type *j*, and *M* is the total number of cell types. Omics layer ASW has a range of 0 to 1, and higher values indicate better mixing.

Graph connectivity (GC) was also used to evaluate the extend of mixing among omics layers and was defined as in a recent benchmark study^73^:

36 $$\begin{array}{*{20}{c}} {{\mathrm{GC}} = \frac{1}{M}\mathop {\sum }\limits_{j = 1}^M \frac{{\left| {{{{\mathrm{LCC}}}}_j} \right|}}{{N_j}}} \end{array}$$

where LCC_*j*_ is the number of cells in largest connected component of the cell *k-*nearest neighbors graph (*K* = 15) for cell type *j*, *N*_*j*_ is the number of cells in cell type *j* and *M* is the total number of cell types. Graph connectivity has a range of 0 to 1, and higher values indicate better mixing.

#### Omics mixing

Seurat alignment score, omics layer ASW and graph connectivity all measure omics mixing of the data integration. Following the procedure from the recent benchmark study^73^, we first conduct min-max scaling for each of the metrics, and then compute the average across the three to summarize them into a single metric representing omics mixing:

37 $$\begin{array}{*{20}{c}} {{\mathrm{omics}}\,{\mathrm{mixing}} = \frac{{{{{\mathrm{scale}}}}\left( {{{{\mathrm{SAS}}}}} \right) + {{{\mathrm{scale}}}}\left( {{{{\mathrm{omics}}}}\,{{{\mathrm{layer}}}}\,{{{\mathrm{ASW}}}}} \right) + {{{\mathrm{scale}}}}\left( {{{{\mathrm{GC}}}}} \right)}}{3}} \end{array}$$

#### Overall integration score

To compute an overall integration score, we use a 6:4 weight between biology conservation and omics mixing, following the recent benchmark study^73^:

38 $$\begin{array}{*{20}{c}} {{\mathrm{overall}}\,{\mathrm{integration}}\,{\mathrm{score}} = 0.6 \times {\mathrm{biology}}\,{\mathrm{conservation}} + 0.4 \times {\mathrm{omics}}\,{\mathrm{mixing}}} \end{array}$$

FOSCTTM^25^ was used to evaluate the single-cell level alignment accuracy. It was computed on two datasets with known cell-to-cell pairings. Suppose that each dataset contains *N* cells, and that the cells are sorted in the same order, that is, the *i*th cell in the first dataset is paired with the *i*th cell in the second dataset. Denote *x* and *y* as the cell embeddings of the first and second dataset, respectively. The FOSCTTM is then defined as:

39 $$\begin{array}{*{20}{c}} {{\mathrm{FOSCTTM}} = \frac{1}{{2N}}\left( {\mathop {\sum }\limits_{i = 1}^N \frac{{n_1^{\left( i \right)}}}{N} + \mathop {\sum }\limits_{i = 1}^N \frac{{n_2^{\left( i \right)}}}{N}} \right)} \end{array}$$

40 $$\begin{array}{*{20}{c}} {n_1^{\left( i \right)} = \left| {\left\{ {j|d\left( {{{{\mathbf{x}}}}_j,{{{\mathbf{y}}}}_i} \right) < d\left( {{{{\mathbf{x}}}}_i,{{{\mathbf{y}}}}_i} \right)} \right\}} \right|} \end{array}$$

41 $$\begin{array}{*{20}{c}} {n_2^{\left( i \right)} = \left| {\left\{ {j|d\left( {{{{\mathbf{x}}}}_i,{{{\mathbf{y}}}}_j} \right) < d\left( {{{{\mathbf{x}}}}_i,{{{\mathbf{y}}}}_i} \right)} \right\}} \right|} \end{array}$$

where $n_1^{\left( i \right)}$ and $n_2^{\left( i \right)}$ are the number of cells in the first and second dataset, respectively, that are closer to the *i*th cell than their true matches in the opposite dataset. *d* is the Euclidean distance. FOSCTTM has a range of 0 to 1, and lower values indicate higher accuracy.

Feature consistency was used to evaluate the consistency of feature embeddings from different models. Since the raw embedding spaces are not directly comparable across models, we defined the consistency as the cross-modal conservation of cosine similarities among features in the same model. Specifically, we first randomly subsample 2,000 features and compute the pairwise cosine similarity among them using feature embeddings from the two compared models. The feature consistency score is then defined as the Pearson’s correlation between the cosine similarities of two models, averaging across four random subsamples. Feature consistency has a range of −1 to 1, and higher values indicate higher consistency.

For the baseline benchmark, each method was run eight times with different random seeds, except for Harmony and bindSC that have deterministic implementations and were run only once. For the guidance corruption benchmark, we removed the specified proportions of existing peak–gene interactions and added equal numbers of nonexistent interactions, so the total number of interactions remained unchanged. Of note, feature conversion was also repeated using the corrupted guidance graphs. The corruption procedure was repeated eight times with different random seeds. For the subsampling benchmark, the scRNA-seq and scATAC-seq cells were subsampled in pairs (so FOSCTTM could still be computed). The subsampling process was also repeated eight times with different random seeds.

For the systematic scalability test (Supplementary Fig. 17a), all methods were run on a Linux workstation with 40 CPU cores (two Intel Xeon Silver 4210 chips), 250 GB of RAM and NVIDIA GeForce RTX 2080 Ti graphical processing units. Only a single graphical processing unit card was used when training GLUE.

### Triple-omics integration

The scRNA-seq and scATAC-seq data were handled as previously described (section Systematic benchmarks). Due to low coverage per single-C site, the snmC-seq data were converted to average methylation levels in gene bodies. The mCH and mCG levels were quantified separately, resulting in two features per gene. The gene methylation levels were normalized by the global methylation level per cell. An initial dimensionality reduction was performed using PCA (section Implementation details). For the triple-omics guidance graph, the mCH and mCG levels were connected to the corresponding genes with negative edges.

The normalized methylation levels were positive, with dropouts corresponding to the genes that were not covered in single cells. As such, we used the zero-inflated log-normal (ZILN) distribution for the data decoder:

42 $$\begin{array}{*{20}{c}} {p\left( {{{{\mathbf{x}}}}_k|{{{\mathbf{u}}}},{{{\mathbf{V}}}};\theta _k} \right) = \mathop {\prod }\limits_{i \in {{{\mathcal{V}}}}_k} {{{\mathrm{ZILN}}}}\left( {{\mathbf{x}_{k}}_{i};{{{\mathbf{\mu }}}}_i,{{{\mathbf{\sigma }}}}_i,{{{\mathbf{\delta }}}}_i} \right)} \end{array}$$

43 $$\begin{array}{*{20}{c}} {{\mathrm{ZILN}}\left( {{\mathbf{x}_{k}}_{i};{{{\mathbf{\mu }}}}_i,{{{\mathbf{\sigma }}}}_i,{{{\mathbf{\delta }}}}_i} \right) = \left\{ {\begin{array}{*{20}{l}} {\frac{{1 - {{{\mathbf{\delta }}}}_i}}{{{\mathbf{x}_{k}}_{i}{{{\mathbf{\sigma }}}}_i\sqrt {2\pi } }}\exp \left( { - \frac{{\left( {\log {\mathbf{x}_{k}}_{i} - {{{\mathbf{\mu }}}}_i} \right)^2}}{{2{{{\mathbf{\sigma }}}}_i^2}}} \right),} \hfill & {{\mathbf{x}_{k}}_{i} > 0} \hfill \\ {{{{\mathbf{\delta }}}}_i,} \hfill & {{\mathbf{x}_{k}}_{i} = 0} \hfill \end{array}} \right.} \end{array}$$

44 $$\begin{array}{*{20}{c}} {{{{\mathbf{\mu }}}}_i = \mathbf{\alpha} \odot {{{\mathbf{V}}}}_k^ \top \mathbf{u} + \mathbf{\beta} } \end{array}$$

where ${{{\mathbf{\mu }}}} \in {\Bbb R}^{\left| {{{{\mathcal{V}}}}_k} \right|},{{{\mathbf{\sigma }}}} \in {\Bbb R}_ + ^{\left| {{{{\mathcal{V}}}}_k} \right|},{{{\mathbf{\delta }}}} \in \left( {0,1} \right)^{\left| {{{{\mathcal{V}}}}_k} \right|}$ are the log-scale mean, log-scale standard deviation and zero-inflation parameters of the zero-inflated log-normal distribution, respectively, and ${{{\mathbf{\alpha }}}} \in {\Bbb R}_ + ^{\left| {{{{\mathcal{V}}}}_k} \right|},{{{\mathbf{\beta }}}} \in {\Bbb R}^{\left| {{{{\mathcal{V}}}}_k} \right|}$ are scaling and bias factors.

To unify the cell type labels, we performed a nearest neighbor-based label transfer with the snmC-seq dataset as a reference. The five nearest neighbors in snmC-seq were identified for each scRNA-seq and scATAC-seq cell in the aligned embedding space, and majority voting was used to determine the transferred label. To verify whether the alignment was correct, we tested for significant overlap in cell type marker genes. The features of all omics layers were first converted to genes. Then, for each omics layer, the cell type markers were identified using the one-versus-rest Wilcoxon rank-sum test with the following criteria: FDR < 0.05 and log fold change >0 for scRNA-seq/scATAC-seq; FDR < 0.05 and log fold change of <0 for snmC-seq. The significance of marker overlap was determined by the three-way Fisher’s exact test^40^.

To perform correlation and regression analysis after the integration, we clustered all cells from the three omics layers using fine-scale *k*-means (*k* = 200). Then, for each omics layer, the cells in each cluster were aggregated into a metacell by summing their expression/accessibility counts or averaging their DNA methylation levels. The metacells were established as paired samples, based on which feature correlation and regression analyses could be conducted.

To integrate the same datasets using online iNMF, we inverted the snmC-seq data via subtracting the data matrix by the largest entry, following the procedure described in the original paper^16^.

### GLUE-based *cis*-regulatory inference

To ensure consistency of cell types, we first selected the overlapping cell types between the 10X Multiome and pcHi-C data. The remaining cell types included T cells, B cells and monocytes. The eQTL data were used as is, because they were not cell type-specific. For scRNA-seq, we selected 6,000 highly variable genes. To capture remote *cis*-regulatory interactions, the base guidance graph was constructed for peak–gene pairs within a distance of 150 kb, using the alternative scheme as described in the section Systematic benchmarks.

To incorporate the regulatory evidence of pcHi-C and eQTL, we anchored all evidence to that between the ATAC peaks and RNA genes. A peak–gene pair was considered supported by pcHi-C if (1) the gene promoter was within 1 kb of a bait fragment, (2) the peak was within 1 kb of an other-end fragment and (3) significant contact was identified between the bait and the other-end fragment in pcHi-C. The pcHi-C-supported peak–gene interactions were weighted by multiplying the promoter-to-bait and the peak-to-other-end power-law weights (above). If a peak–gene pair was supported by multiple pcHi-C contacts, the weights were summed and clipped to a maximum of 1. A peak–gene pair was considered supported by eQTL if (1) the peak overlapped an eQTL locus and (2) the locus was associated with the expression of the gene. The eQTL-supported peak–gene interactions were assigned weights of 1. The composite guidance graph was constructed by adding the pcHi-C- and eQTL-supported interactions to the previous distance-based interactions, allowing for multi-edges.

For regulatory inference, only peak–gene pairs within 150 kb in distance were considered. The GLUE training process was repeated four times with different random seeds. For each repeat, the peak–gene regulatory score was computed as the cosine similarity between the feature embeddings. The final regulatory inference was obtained by averaging the regulatory scores across the four repeats. To evaluate the significance of the regulatory scores, we compared the scores to a NULL distribution obtained via randomly shuffled feature embeddings and computed empirical *P* values as the probability of getting more extreme scores in the NULL distribution. Finally, we compute FDR of regulatory inference based on the *P* values using the Benjamini–Hochberg procedure. For *cis*-regulatory inference using LASSO, we used hyperparameter *α* = 0.01, which was optimized for area under the receiver operating characteristic curves of pcHi-C and eQTL prediction.

### TF-target gene regulatory inference

We used the SCENIC workflow^76^ to construct a TF-gene regulatory network from the inferred peak–gene regulatory interactions. Briefly, the SCENIC workflow first constructs a gene coexpression network based on the scRNA-seq data, and then uses external *cis*-regulatory evidence to filter out false positives. SCENIC accepts *cis*-regulatory evidence in the form of gene rankings per TF, that is, genes with higher TF enrichment levels in their regulatory regions are ranked higher. To construct the rankings based on our inferred peak–gene interactions, we first overlapped the ENCODE TF chromatin immunoprecipitation (ChIP) peaks^77^ with the ATAC peaks and counted the number of ChIP peaks for each TF in each ATAC peak. Since different genes can have different numbers of connected ATAC peaks, and the ATAC peaks vary in length (longer peaks can contain more ChIP peaks by chance), we devised a sampling-based approach to evaluate TF enrichment. Specifically, for each gene, we randomly sampled 1,000 sets of ATAC peaks that matched the connected ATAC peaks in both number and length distribution. We counted the numbers of TF ChIP peaks in these random ATAC peaks as null distributions. For each TF in each gene, an empirical *P* value could then be computed by comparing the observed number of ChIP peaks to the null distribution. Finally, we ranked the genes by the empirical *P* values for each TF, producing the *cis*-regulatory rankings used by SCENIC. Since peak–gene-based inference is mainly focused on remote regulatory regions, proximal promoters could be missed. As such, we provided SCENIC with both the above peak-based and proximal promoter-based *cis*-regulatory rankings.

### Integration for the human multi-omics atlas

The scRNA-seq and scATAC-seq atlases have highly unbalanced cell type compositions, which are primarily caused by differences in organ sampling sizes (Supplementary Fig. 17b). Although cell types are unknown during real-world analyses, organ sources are typically available and can be used to help balance the integration process. To perform organ-balanced data preprocessing, we first subsampled each omics layer to match the organ compositions. For the scRNA-seq data, 4,000 highly variable genes were selected using the organ-balanced subsample. Then, for the initial dimensionality reduction, we fitted PCA (scRNA-seq) and LSI (scATAC-seq) on the organ-balanced subsample and applied the projection to the full data. The PCA/LSI coordinates were used as the first transformation layer in the GLUE data encoders (section Implementation details), as well as for metacell aggregation (below). The guidance graph was constructed as described previously (section Systematic benchmarks).

The two atlases consist of large numbers of cells but with low coverage per cell. To alleviate dropout and increase the training speed simultaneously, we used a metacell aggregation strategy during pretraining. Specifically, in the pretraining stage, we clustered the cells in each omics layer using fine-scaled *k*-means (*k* = 100,000 for scRNA-seq and *k* = 40,000 for scATAC-seq). To balance the organ compositions at the same time, *k*-means centroids were fitted on the previous organ-balanced subsample and then applied to the full data. The cells in each *k*-means cluster were aggregated into a metacell by summing their expression/accessibility counts and averaging their PCA/LSI coordinates. GLUE was then pretrained on the aggregated metacells with additive noise, which roughly oriented the cell embeddings but did not actually align them (section Weighted adversarial alignment). To better use the large data size, the hidden layer dimensionality was doubled to 512 from the default 256. In the second stage, GLUE was fine-tuned on the full single-cell data with the balancing weight estimated as described in the section Weighted adversarial alignment. No metacell aggregation was used when comparing the scalability of different methods (Supplementary Fig. 17a).

For a comparison with other integration methods, we also tried online iNMF and Seurat v.3. Online iNMF was the only other method that could scale to millions of cells, so we applied it to the full dataset. On the other hand, Seurat v.3 showed the second-best accuracy in our previous benchmark. We also managed to apply it to the aggregated data used in the first stage of GLUE training, due to the fact that Seurat v.3 could not scale to the full dataset (Supplementary Fig. 17a). Label transfer was performed using the same procedure as in the triple-omics case, except that we used majority voting in 50 nearest neighbors.

### Reporting Summary

Further information on research design is available in the Nature Research Reporting Summary linked to this article.

## Online content

Any methods, additional references, Nature Research reporting summaries, source data, extended data, supplementary information, acknowledgements, peer review information; details of author contributions and competing interests; and statements of data and code availability are available at 10.1038/s41587-022-01284-4.

## Supplementary information

Supplementary Information Supplementary Figs. 1–23 and Table 1.

Reporting Summary

Supplementary Data 1 Detailed benchmarking data.

Supplementary Data 2 Regulatory interactions in the GLUE-derived TF-target gene network.

## Data Availability

All datasets used in this study are already published and were obtained from public data repositories. See Supplementary Table 1 for detailed information on single-cell omics datasets used in this study, including access codes and URLs. For regulatory inference and evaluation, the pcHi-C data was obtained from supplementary file of the original publication (https://www.sciencedirect.com/science/article/pii/S0092867416313228), eQTL data from GTEx v8 (https://www.gtexportal.org/home/datasets), TF ChIP–seq data from ENCODE data portal (https://www.encodeproject.org/) and TRRUST v2 database from the official website (https://www.grnpedia.org/trrust/downloadnetwork.php). All benchmarking source data are available in Supplementary Data 1.

## Extended data

are available for this paper at 10.1038/s41587-022-01284-4.

## Code availability

The GLUE framework was implemented in the ‘scglue’ Python package, which is available at https://github.com/gao-lab/GLUE. For reproducibility, the scripts for all benchmarks and case studies were assembled using Snakemake (v.6.12.3), which is also available in the above repository.
