# Supplementary material for: Multi-omics single-cell data integration and regulatory inference with graph-linked embedding
Source: Nat Biotechnol. 2022 May 2;40(10):1458–66. doi: 10.1038/s41587-022-01284-4 (PMC9546775; doi:10.1038/s41587-022-01284-4)
Supplement: Supplementary file 1 — Supplementary Figs. 1–23 and Table 1. [file 41587_2022_1284_MOESM1_ESM.pdf]

nature portfolio

https://doi.org/10.1038/s41587-022-01284-4

Supplementary information

Multi-omics single-cell data integration and regulatory inference with graph-linked embedding

In the format provided by the authors and unedited

Supplementary Information

Multi-omics single-cell data integration and regulatory inference with graph-linked embedding

Cao et al.

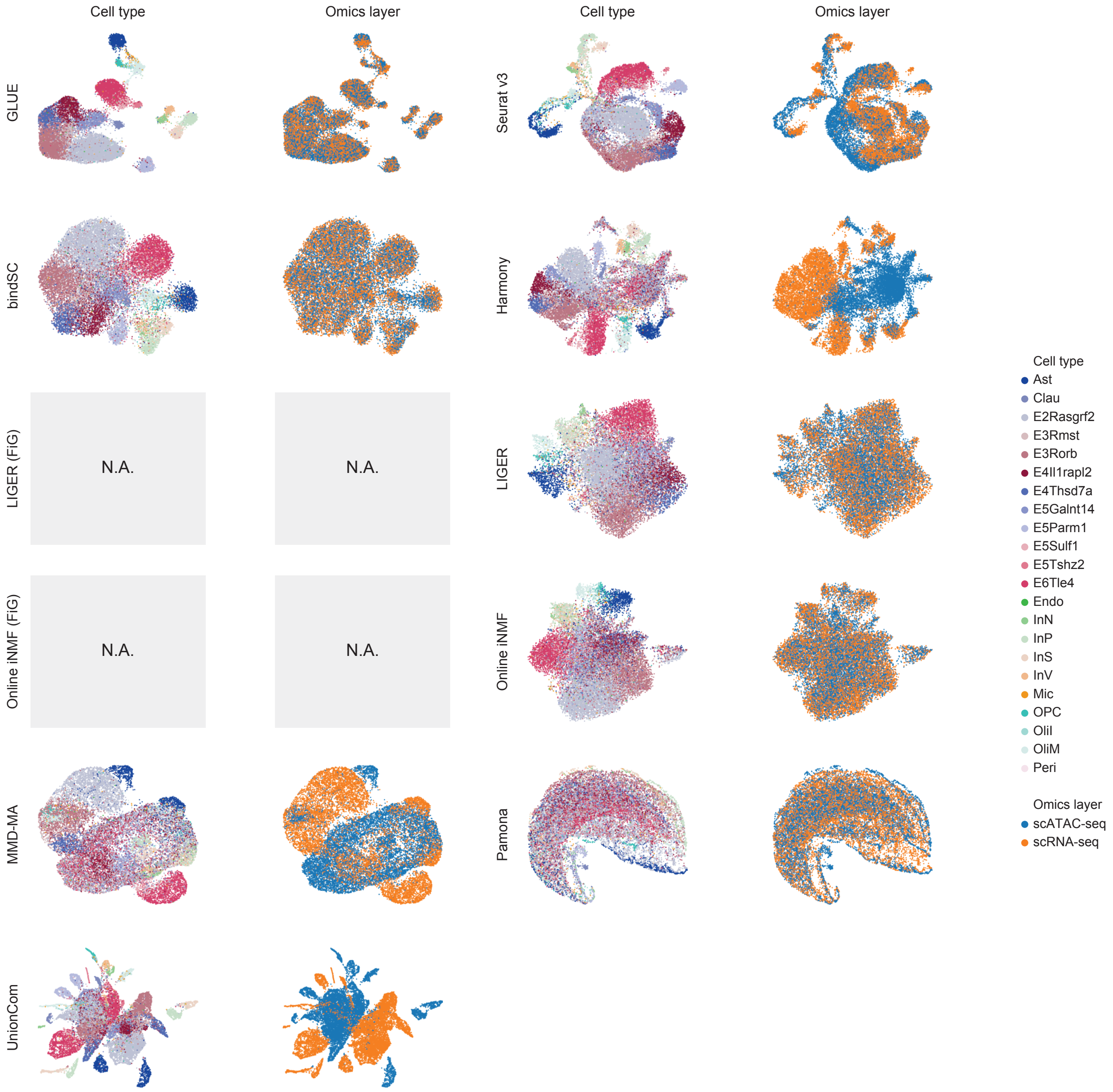

Supplementary Fig. 1 UMAP visualizations of the cell embeddings in the SNARE-seq dataset aligned with different integration methods.
Online iNMF and LIGER could not run with FiG conversion because raw ATAC fragment file was not available.

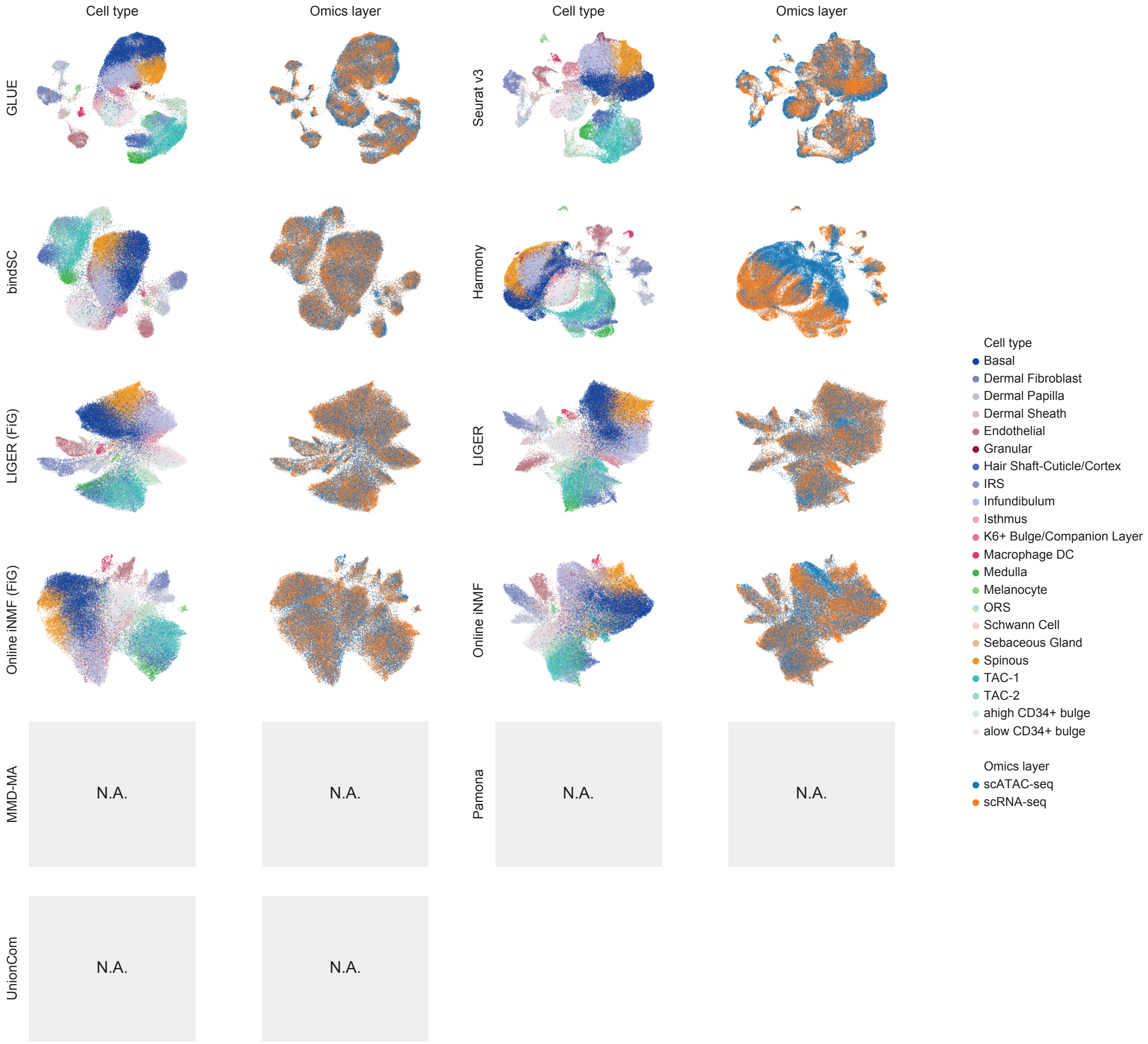

Supplementary Fig. 2 UMAP visualizations of the cell embeddings in the SHARE-seq dataset aligned with different integration methods.

UnionCom, Pamona, and MMD-MA failed to run because of memory overflow.

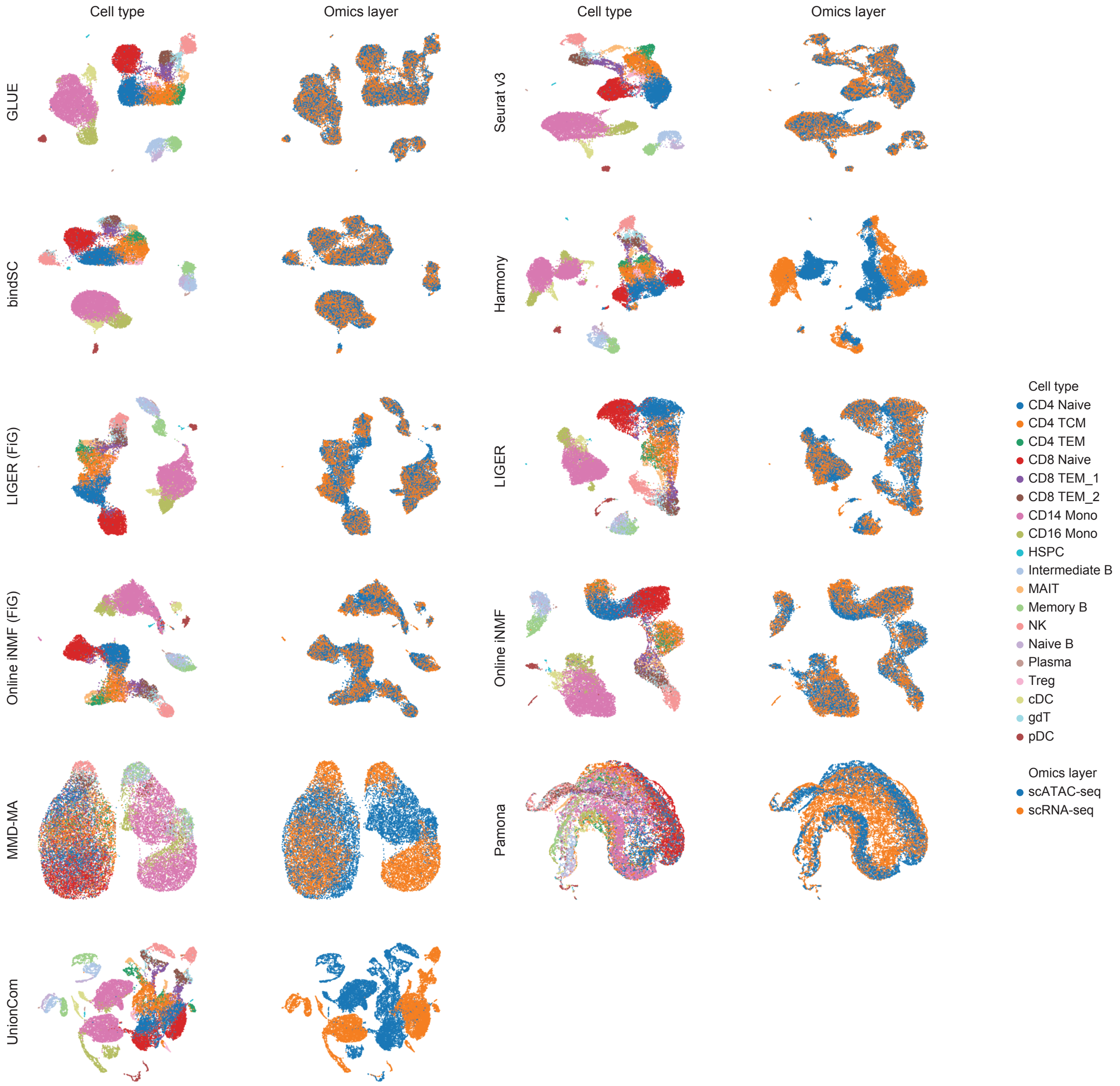

Supplementary Fig. 3 UMAP visualizations of the cell embeddings in the 10x Multiome dataset aligned with different integration methods.

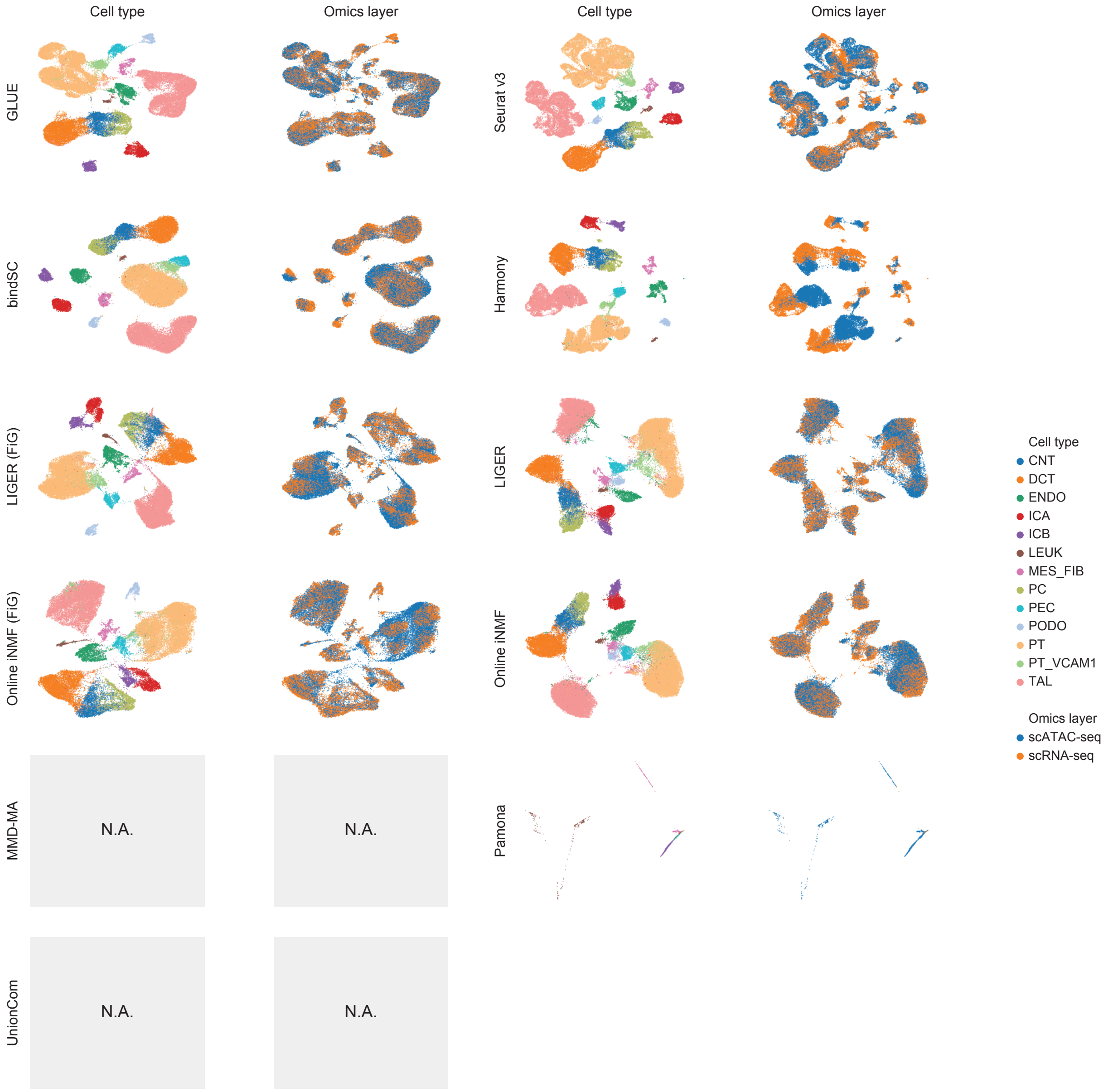

Supplementary Fig. 4 UMAP visualizations of the cell embeddings in the Nephron dataset aligned with different integration methods.
UnionCom and MMD-MA failed to run because of memory overflow.

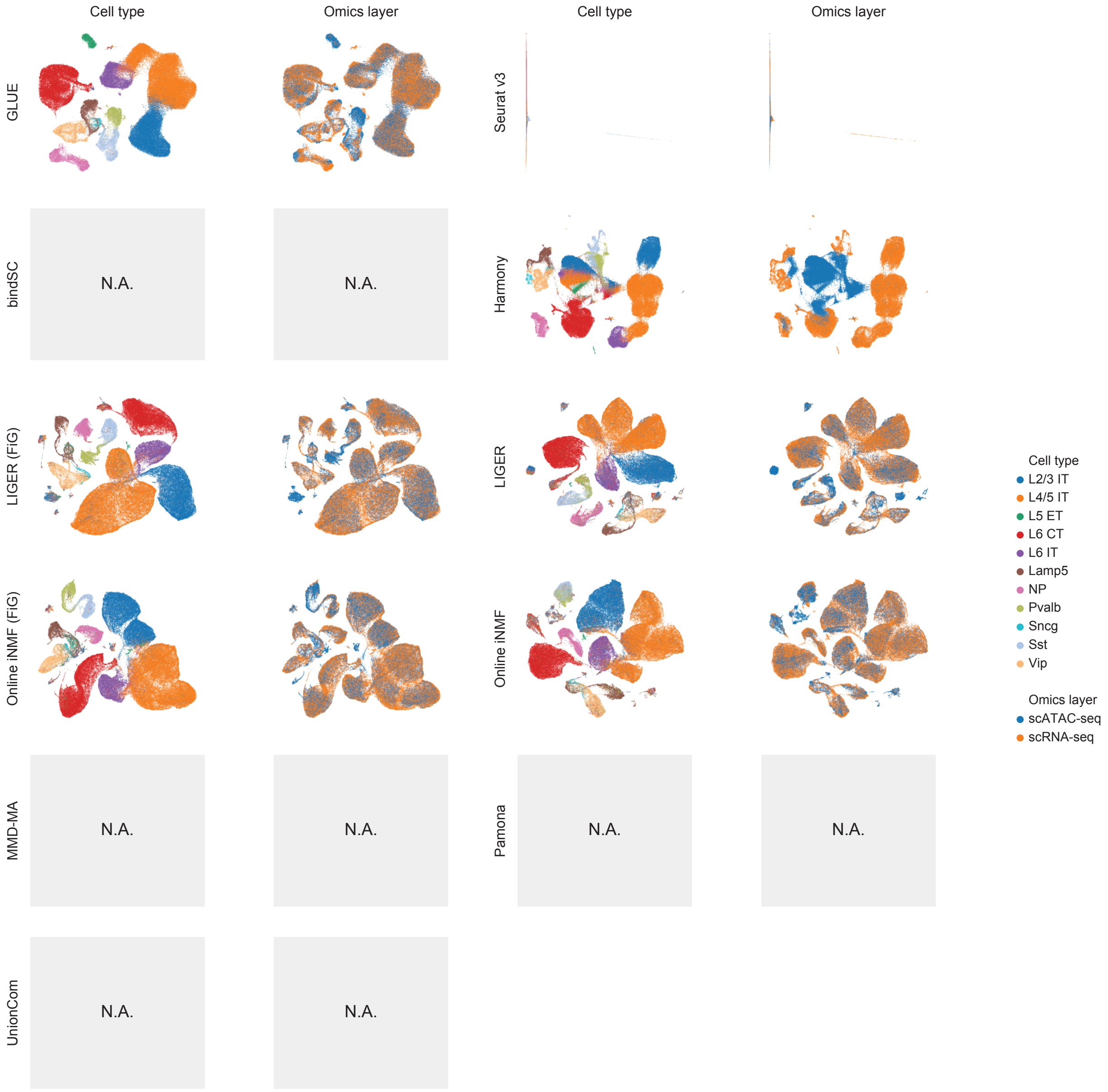

Supplementary Fig. 5 UMAP visualizations of the cell embeddings in the Mop dataset aligned with different integration methods.
UnionCom, Pamona, MMD-MA and bindSC failed to run because of memory overflow.

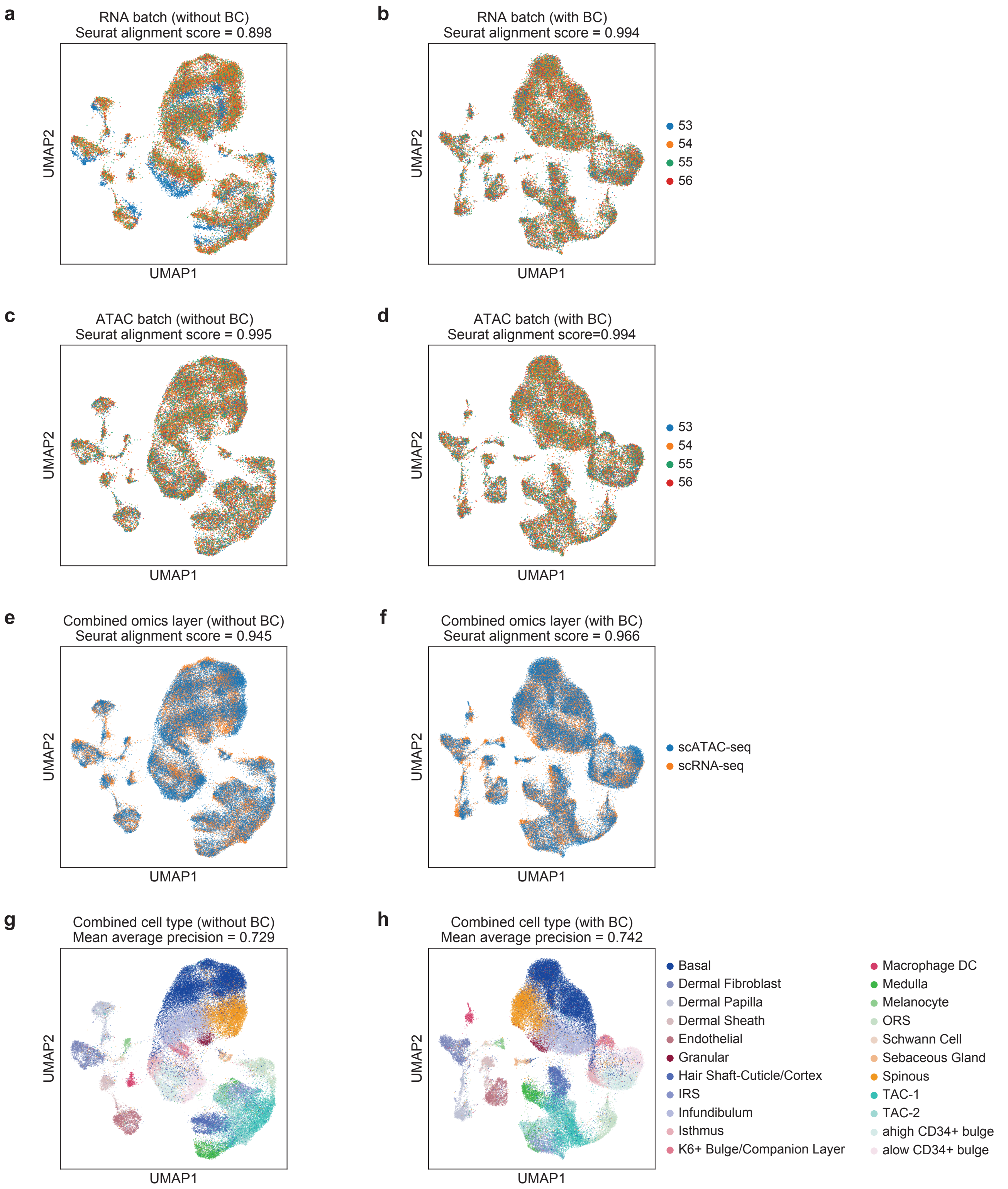

Supplementary Fig. 6 GLUE batch effect correction in the SHARE-seq dataset.
a, **b**, Batch-colored UMAP visualization of GLUE-integrated scRNA-seq cell embeddings **a**, without, and **b**, with batch correction. **c**, **d**, Cell type-colored UMAP visualization of GLUE-integrated scATAC-seq cell embeddings **c**, without, and **d**, with batch correction. **e**, **f**, Omics layer-colored UMAP visualization of all cell embeddings **e**, without, and **f**, with batch correction. **g**, **h**, Cell type-colored UMAP visualization of all cell embeddings **g**, without, and **h**, with batch correction. “BC” stands for batch correction.

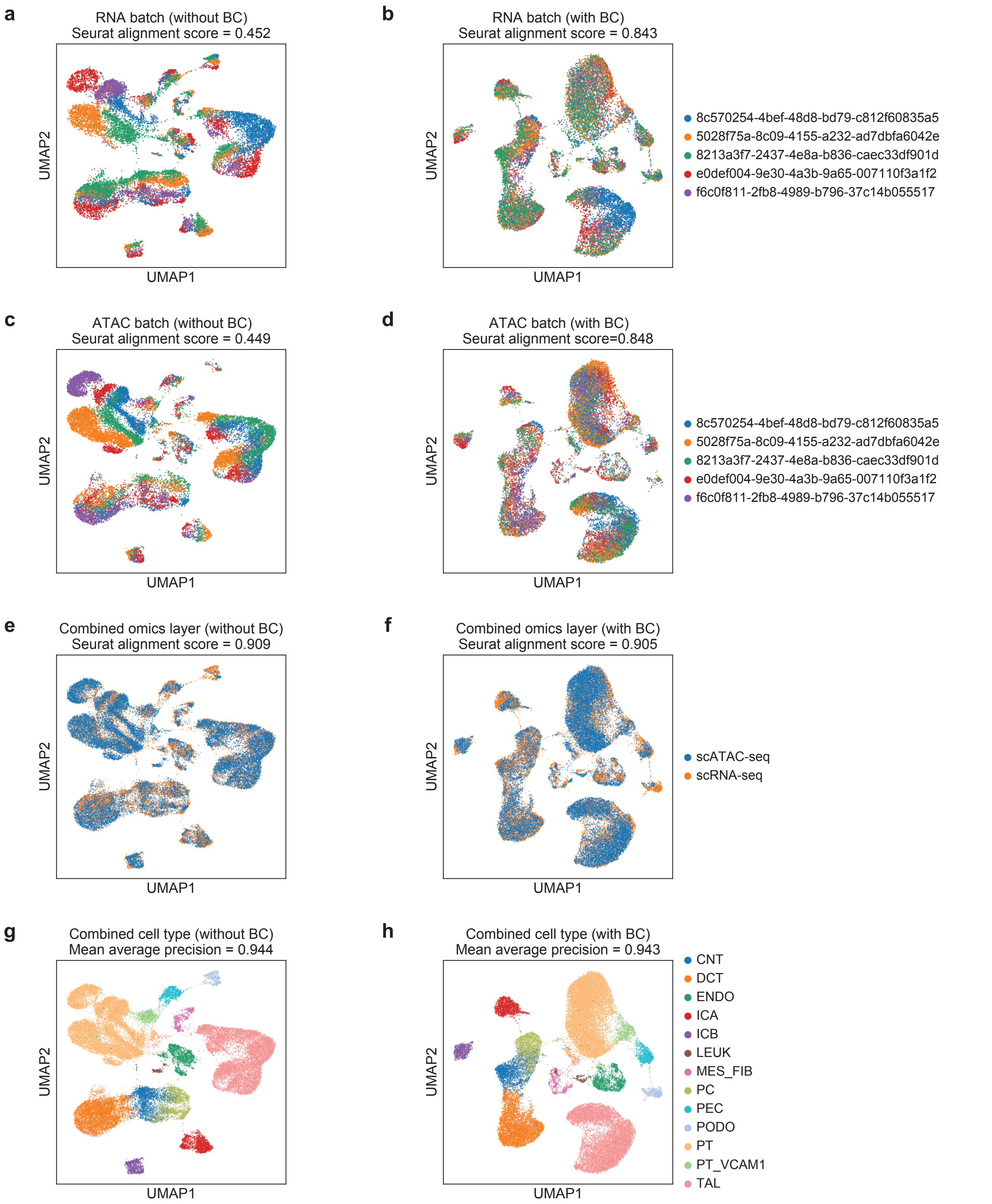

Supplementary Fig. 7 Effect of batch effect correction in the Nephron dataset.
a, **b**, Batch-colored UMAP visualization of GLUE-integrated scRNA-seq cell embeddings **a**, without, and **b**, with batch correction. **c**, **d**, Cell type-colored UMAP visualization of GLUE-integrated scATAC-seq cell embeddings **c**, without, and **d**, with batch correction. **e**, **f**, Omics layer-colored UMAP visualization of all cell embeddings **e**, without, and **f**, with batch correction. **g**, **h**, Cell type-colored UMAP visualization of all cell embeddings **g**, without, and **h**, with batch correction. “BC” stands for batch correction.

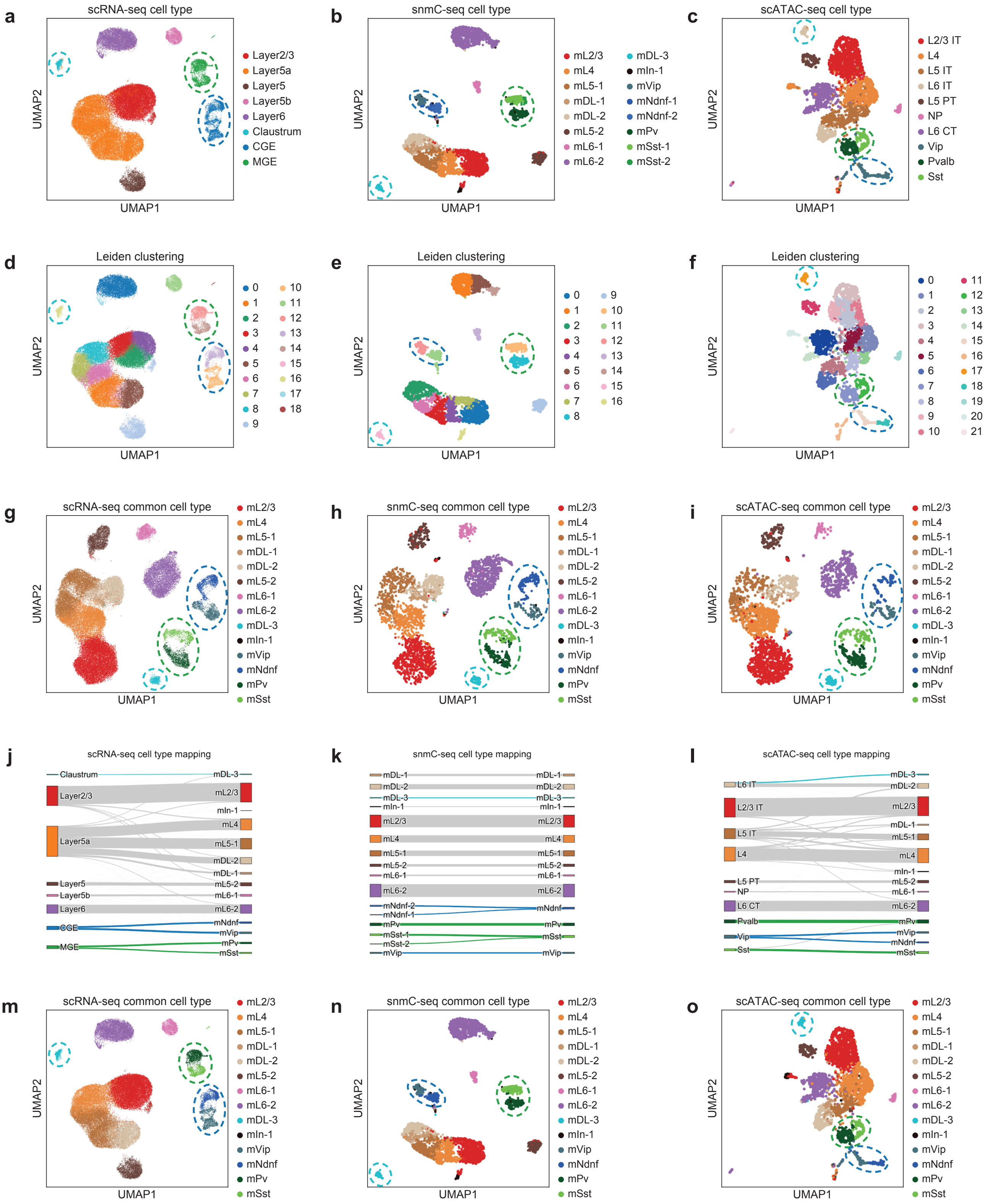
a
scRNA-seq cell type
UMAP2
UMAP1
Layer2/3
Layer5a
Layer5
Layer5b
Layer6
Claustrum
CGE
MGE
b
snmC-seq cell type
mL2/3
mL4
mL5-1
mDL-1
mDL-2
mL5-2
mL6-1
mL6-2
mDL-3
mIn-1
mVip
mNdnf-1
mNdnf-2
mPv
mSst-1
mSst-2
c
scATAC-seq cell type
L2/3 IT
L4
L5 IT
L6 IT
L5 PT
NP
L6 CT
Vip
Pvalb
Sst
d
Leiden clustering
e
Leiden clustering
f
Leiden clustering
g
scRNA-seq common cell type
h
snmC-seq common cell type
i
scATAC-seq common cell type
mL2/3
mL4
mL5-1
mDL-1
mDL-2
mL5-2
mL6-1
mL6-2
mDL-3
mIn-1
mVip
mNdnf
mPv
mSst
j
scRNA-seq cell type mapping
k
snmC-seq cell type mapping
l
scATAC-seq cell type mapping
m
scRNA-seq common cell type
n
snmC-seq common cell type
o
scATAC-seq common cell type

Supplementary Fig. 8 Triple-omics alignment and label transfer.
a-**c**, UMAP visualization of single-layer representations colored by original cell types for **a**, scRNA-seq (via PCA), **b**, snmC-seq (via PCA), and **c**, scATAC-seq (via LSI). **d**-**f**, UMAP visualization of single-layer cell embeddings colored by Leiden clustering for **d**, scRNA-seq, **e**, snmC-seq, and **f**, scATAC-seq. Note that the MGE, CGE types in scRNA-seq, and the L6-IT, Pvalb types in scATAC-seq could be further clustered into two subtypes each. **g**-**i**, UMAP visualizations of GLUE-integrated cell embeddings colored by the unified cell types (labels transferred from snmC-seq) for **g**, scRNA-seq, **h**, snmC-seq, and **i**, scATAC-seq. **j**-**l**, Alluvial diagrams comparing the original cell types and unified cell types in **j**, scRNA-seq, **k**, snmC-seq, and **l**, scATAC-seq. The original cell types are to the left, and the unified cell types are to the right. Cells relabeled to "mPv" and "mSst" are highlighted with green flows. Cells relabeled to "mNdnf" and "mVip" are highlighted with dark blue flows. Cells relabeled to "mDL-3" are highlighted with light blue flows. **m**-**o**, UMAP visualization of single-layer cell embeddings colored by the unified cell types for **m**, scRNA-seq, **n**, snmC-seq, and **o**, scATAC-seq.

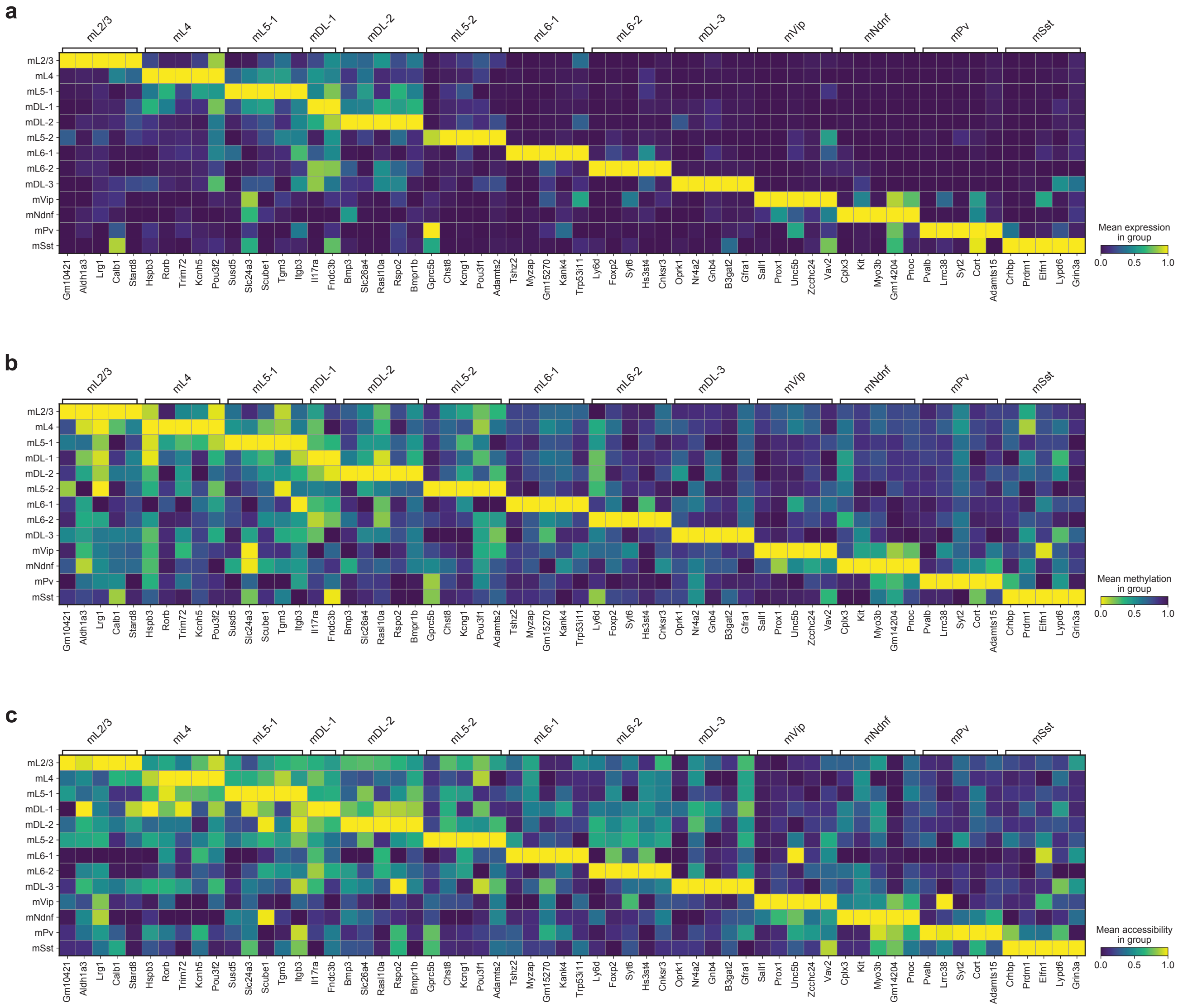

Supplementary Fig. 9 Consensus cell type markers across three omics layers.

a, Expression in scRNA-seq. **b**, Gene body DNA methylation in snmC-seq. **c**, Chromatin accessibility in scATAC-seq. Note that an inverted color bar is used for DNA methylation.

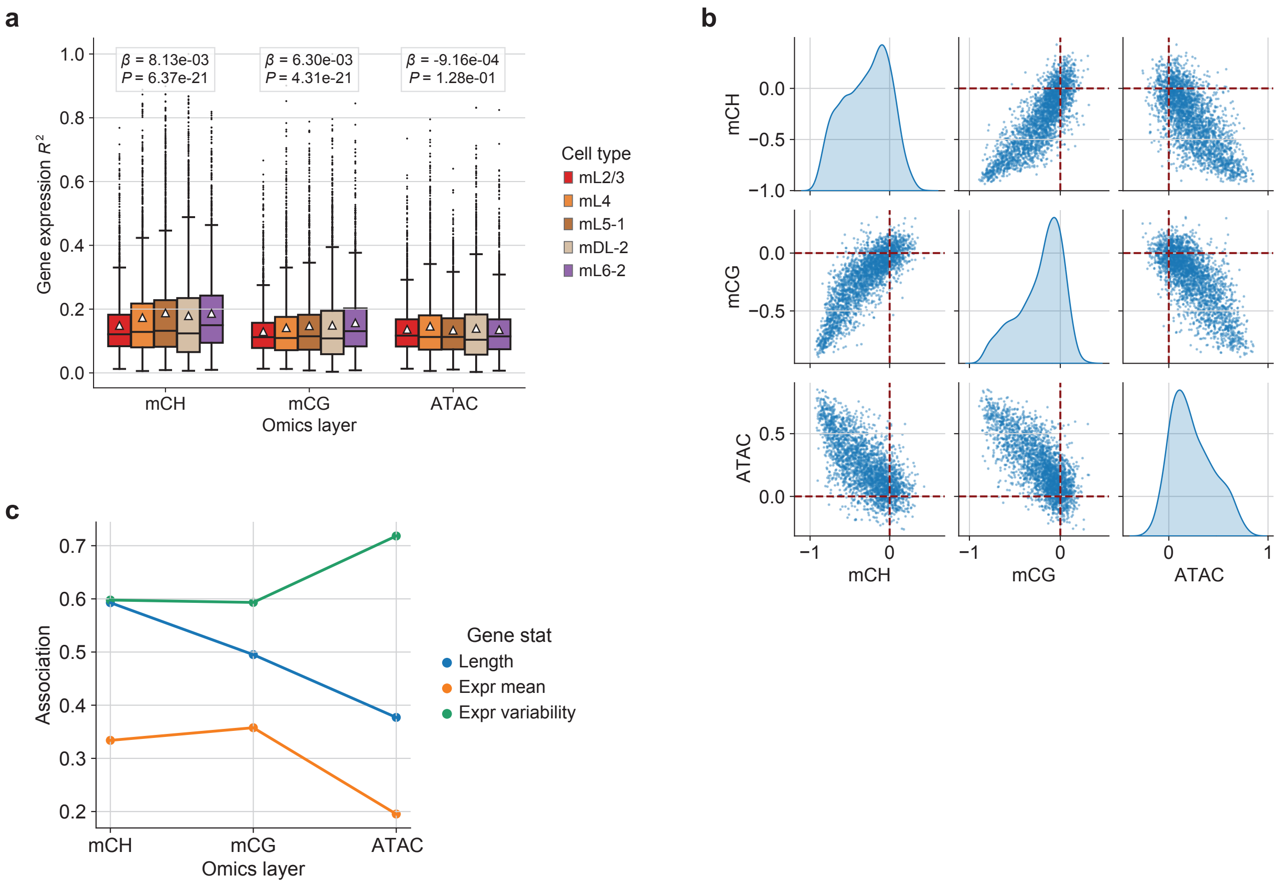

Supplementary Fig. 10 Epigenetic contributions to gene expression.

a, Coefficient of determination (R^2) for predicting gene expression based on each epigenetic layer in different cell types (n=2,677 highly variable genes common to all three omics layers). The box plots indicate the medians (centerlines), means (triangles), 1st and 3rd quartiles (bounds of boxes), and 1.5× interquartile range (whiskers). Above each epigenetic layer, the linear regression slope (β) and its P value (two-tailed t-test) are displayed, with the cell type as the regressor. **b**, Pearson's correlations between gene expression and different epigenetic states. **c**, Associations (defined as the absolute values of Pearson's correlations) between the correlations in **b** and different gene characteristics.

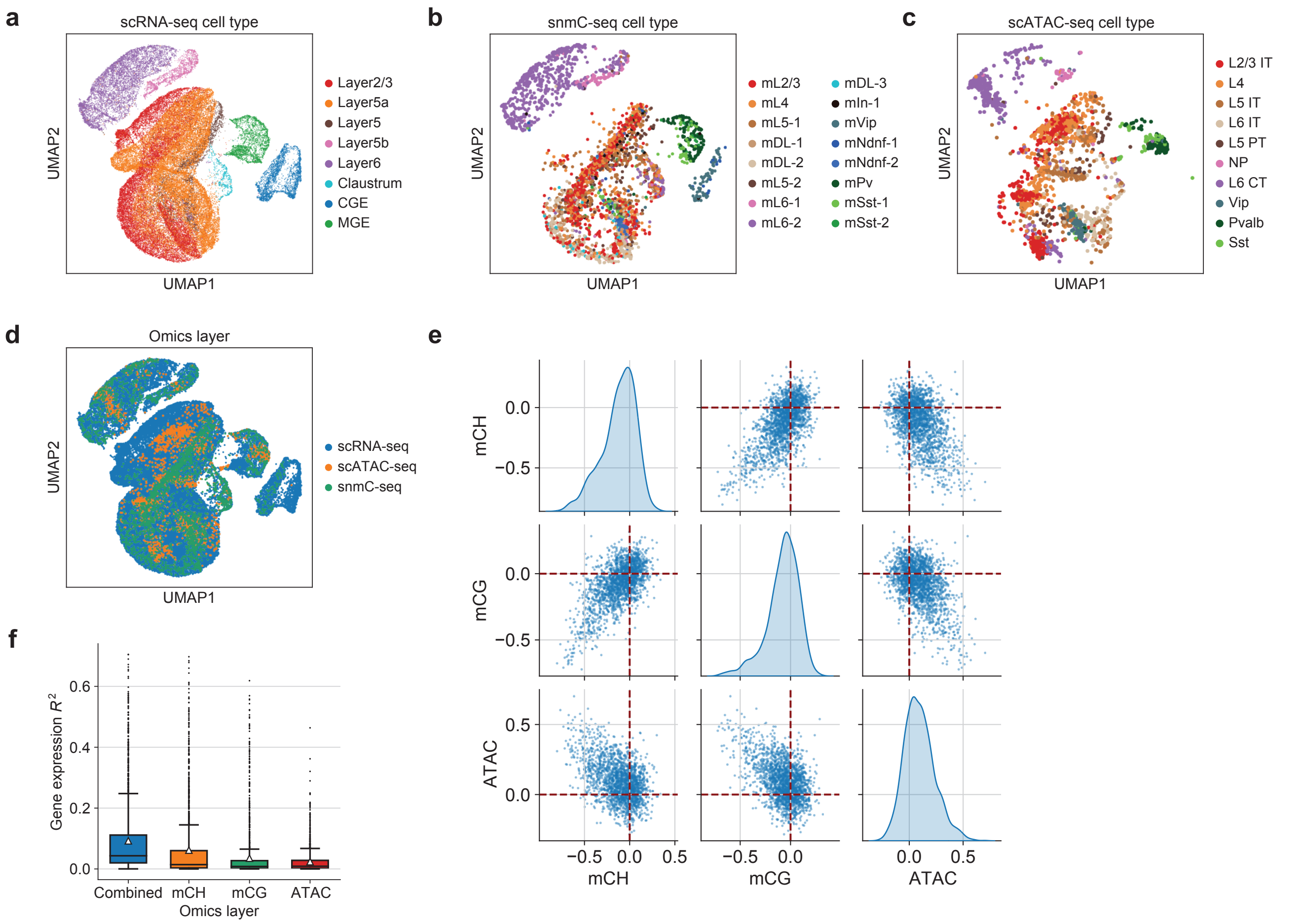

Supplementary Fig. 11 Triple-omics alignment using online iNMF.

a-**c**, UMAP visualizations of the integrated cell embeddings for **a**, scRNA-seq, **b**, snmC-seq, and **c**, scATAC-seq, colored by the original cell types. **d**, UMAP visualizations of the integrated cell embeddings for all cells, colored by omics layers. **e**, Pearson's correlations between gene expression and different epigenetic states. **f**, Coefficient of determination (R^2) for predicting gene expression based on each epigenetic layer as well as the combination of all layers (n=1,933 highly variable genes common to all three omics layers). The box plots indicate the medians (centerlines), means (triangles), 1st and 3rd quartiles (bounds of boxes), and 1.5× interquartile range (whiskers).

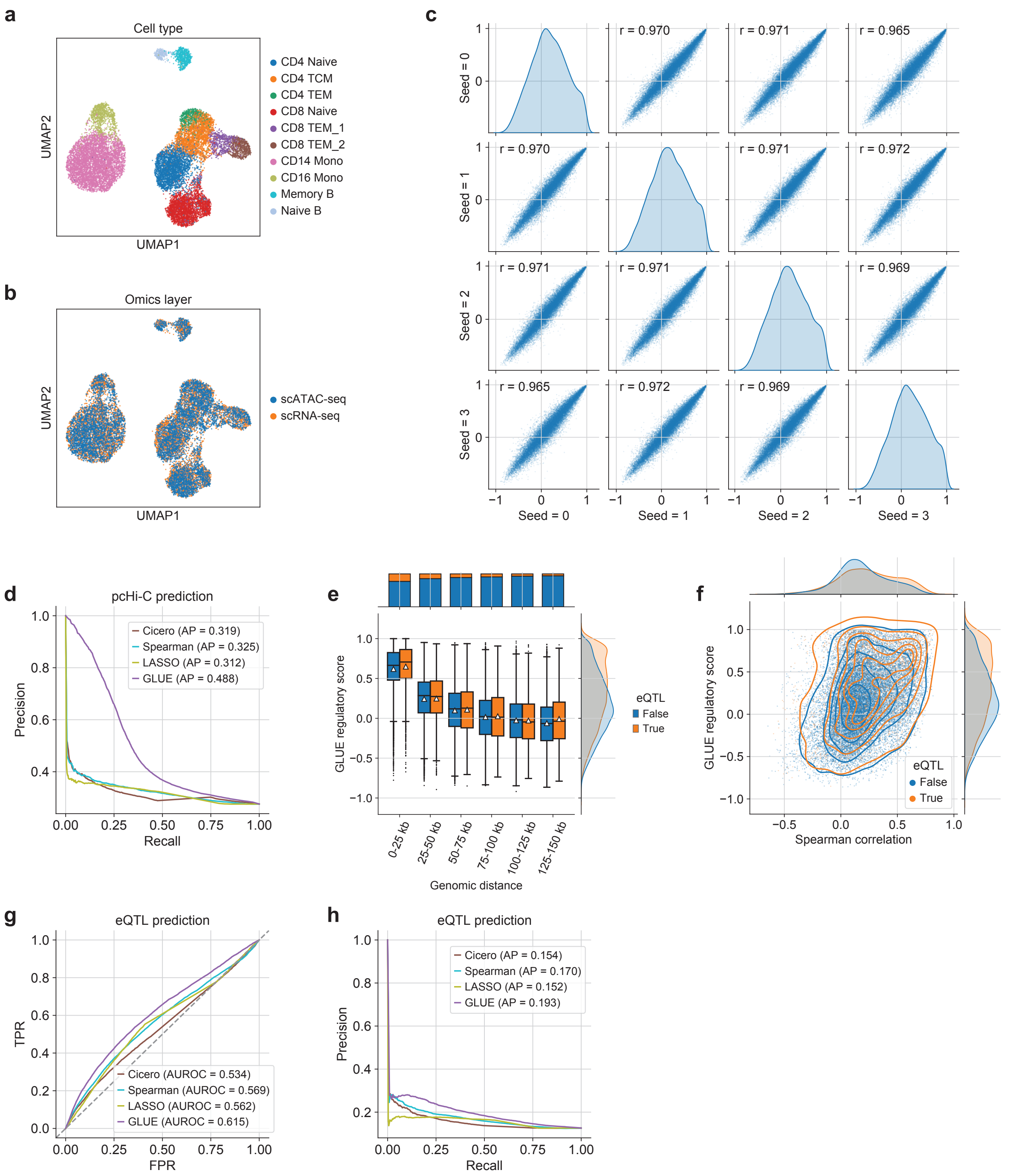

Supplementary Fig. 12 GLUE regulatory inference using distance-based power-law interactions as guidance.

a, **b**, UMAP visualizations of the integrated cell embeddings colored by **a**, cell types, and **b**, omics layers. **c**, Pearson's correlation coefficients for the GLUE regulatory scores across different random seeds. **d**, PRC (precision recall curve) for predicting pcHi-C interactions based on different peak-gene association scores. AP (average precision) quantifies the area under the PRC curve. **e**, GLUE regulatory scores for peak-gene pairs across different genomic ranges, grouped by whether they had eQTL support. The box plots indicate the medians (centerlines), means (triangles), 1st and 3rd quartiles (bounds of boxes), and 1.5× interquartile range (whiskers). **f**, Comparison between the GLUE regulatory scores and the empirical peak-gene correlations computed on paired cells. Peak-gene pairs are colored by whether they had eQTL support. **g**, ROC curves for predicting eQTL interactions based on different peak-gene association scores. **h**, PRC for predicting eQTL interactions based on different peak-gene association scores.

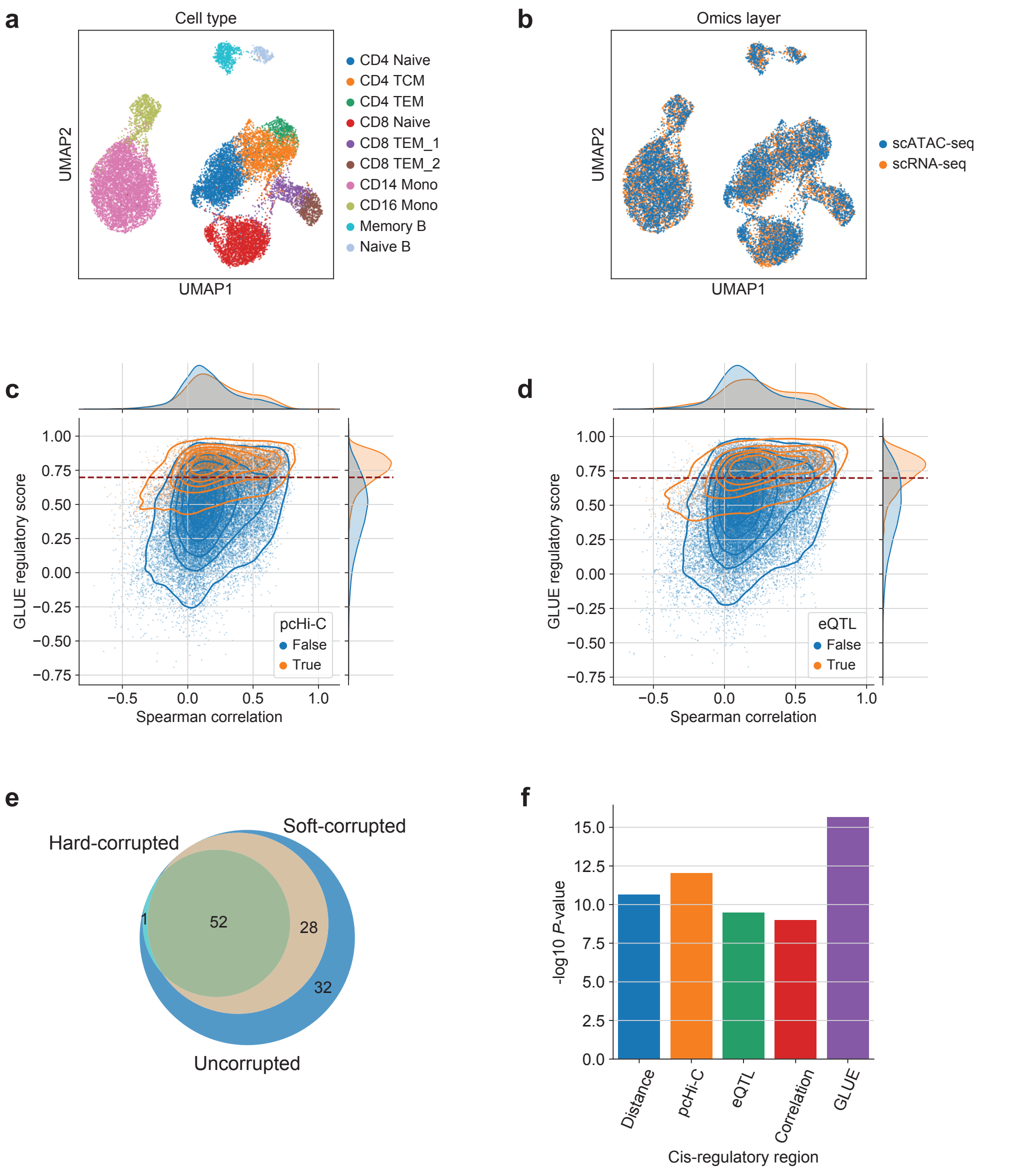

Supplementary Fig. 13 GLUE regulatory inference using a combination of distance-based, eQTL and pcHi-C interactions as guidance.

a, **b**, UMAP visualizations of the integrated cell embeddings colored by **a**, cell types, and **b**, omics layers. **c**, **d**, Comparison between the GLUE regulatory scores and the empirical peak-gene correlations computed on paired cells. The peak-gene pairs are colored by whether they had **c**, pcHi-C support or **d**, eQTL support. Red dashed lines indicate the 75th percentile of the GLUE regulatory scores, which was used as a cutoff. The GLUE-identified interactions mostly exhibited positive empirical correlations and covered the majority of pcHi-C and eQTL support, while the selection of peak-gene pairs based solely on empirical correlation would lead to much lower external support. **e**, Numbers of recovered peak-gene associations among 124 high-confidence ones. 113 could be recovered when the guidance graph was not corrupted. 70.8% (80 out of 113) could be recovered with a soft corrupted graph (pcHi-C and eQTL-based links between high-confidence pairs removed, leaving distance-based links unchanged). 46.9% (53 out of 113) could be recovered with a hard corrupted graph (all links between high-confidence pairs removed, including distance-based links). **f**, Consistency between the TF-target gene networks constructed with different peak-gene association methods and the manually curated connections in the TRRUST v2 database (Fisher's exact test).

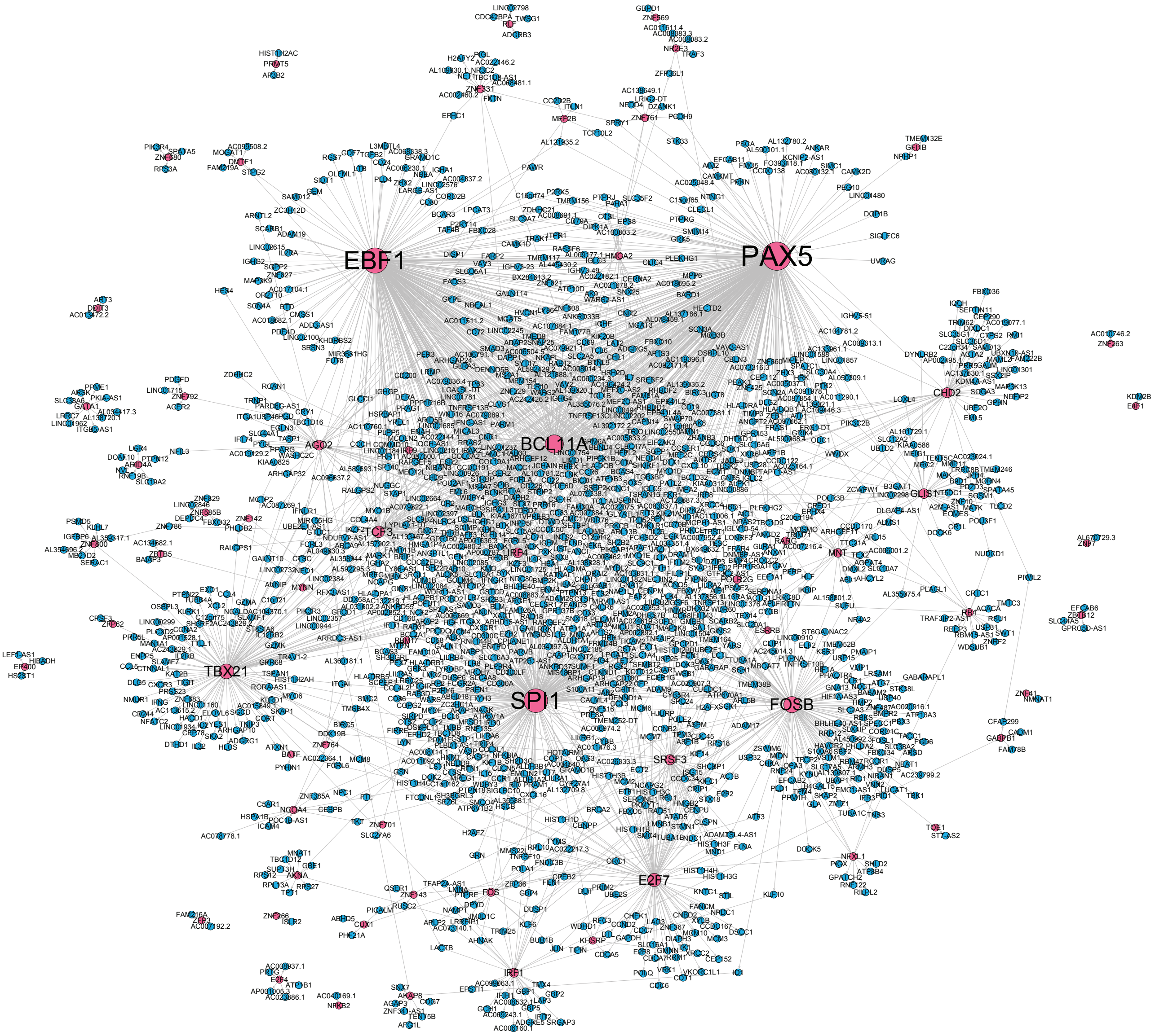

Supplementary Fig. 14 Inferred TF-target gene regulatory network in PBMC.
TFs are colored in pink, and target genes are colored in blue. Node sizes are proportional to their degrees in the graph.

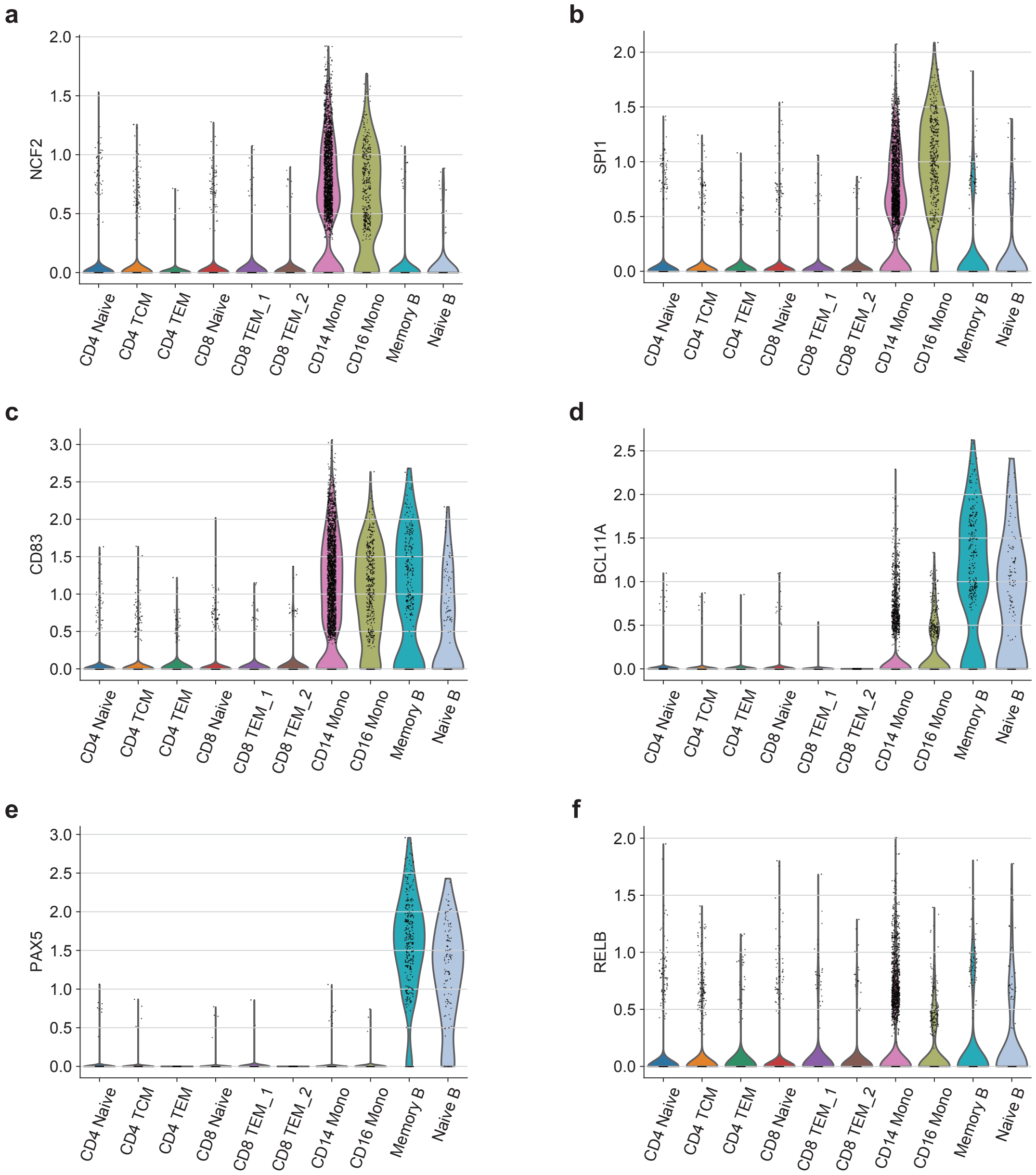

Supplementary Fig. 15 TF-target gene expression levels in different cell types.
a, **b**, Expression levels of **a**, *NCF2*, and its regulator **b**, *SPI1* in different cell types. **c-f**, Expression levels of **c**, *CD83*, and its inferred regulators **d**, *BCL11A*, **e**, *PAX5*, and **f**, *RELB* in different cell types.

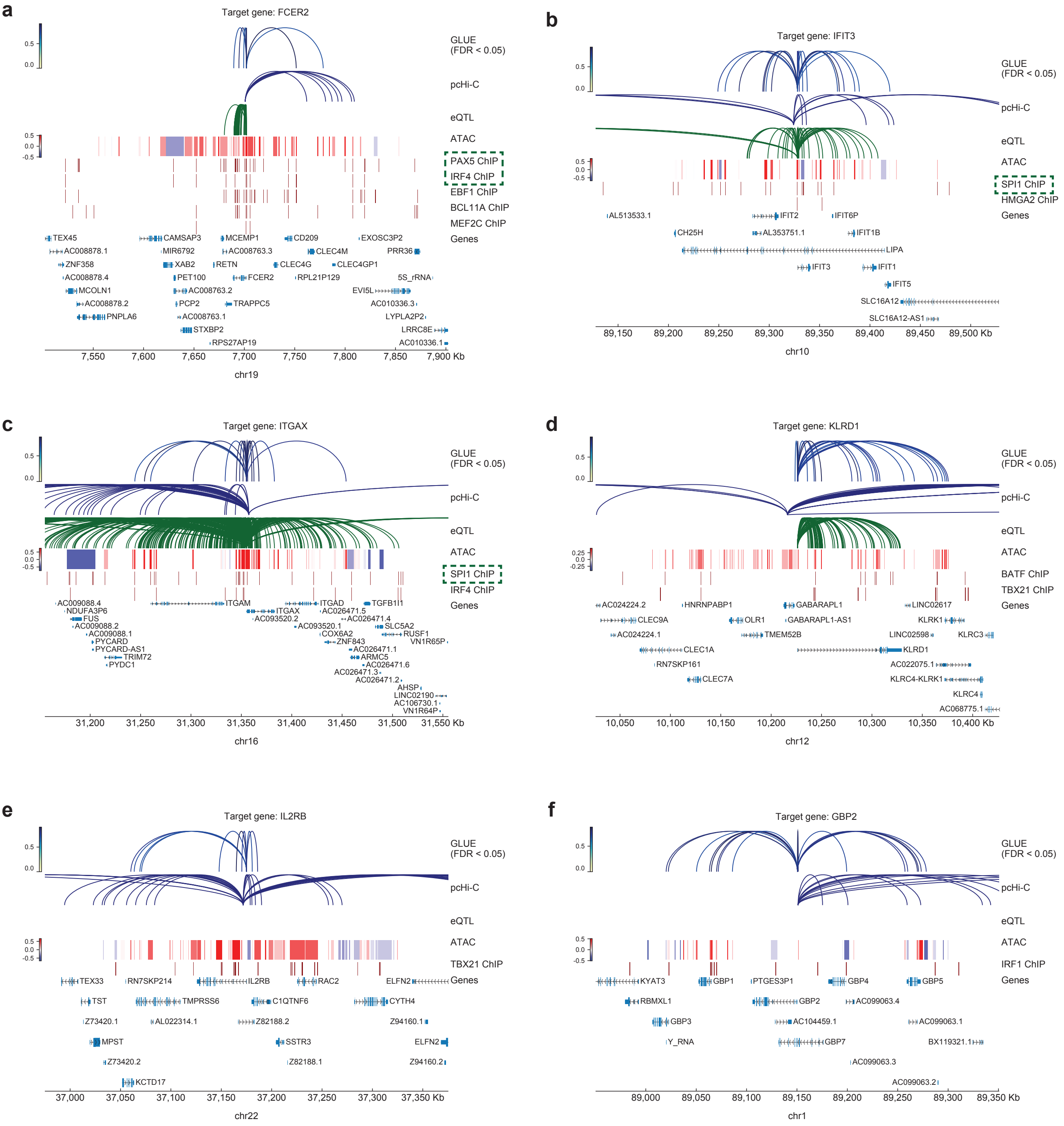

Supplementary Fig. 16 Regulatory inference and evidence per gene.
GLUE-identified cis-regulatory interactions for **a**, *FCER2*, **b**, *IFIT3*, **c**, *ITGAX*, **d**, *KLRD1*, **e**, *IL2RB*, and **f**, *GBP2*, along with individual pieces of regulatory evidence. For each gene, the ChIP-seq tracks correspond to inferred TF regulators. Known regulators are highlighted with green boxes.

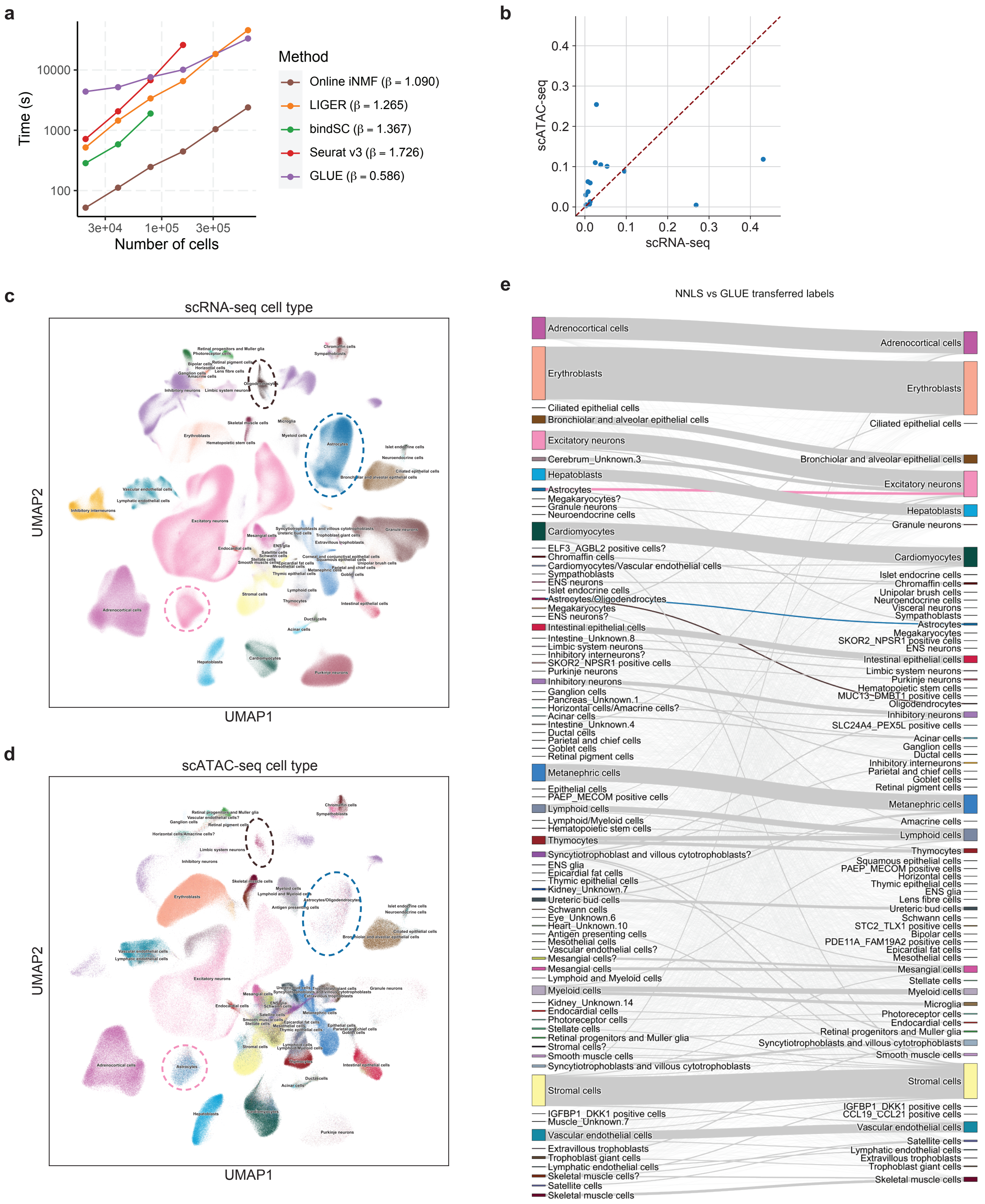

a
Time (s)
Number of cells
Method
Online iNMF (β = 1.090)
LIGER (β = 1.265)
bindSC (β = 1.367)
Seurat v3 (β = 1.726)
GLUE (β = 0.586)
b
scATAC-seq
scRNA-seq
c
scRNA-seq cell type
UMAP1
UMAP2
d
scATAC-seq cell type
e
NNLS vs GLUE transferred labels

Supplementary Fig. 17 Scalability benchmarking and integration of a multi-omics human cell atlas.

a, Time costs of different methods on subsampled atlases of varying sizes. The time costs and cell numbers are both plotted in log-scale, so the slope β represents the exponent in original scale, i.e., $\beta = 2$ represents quadratic scalability, $\beta = 1$ represents linear scalability, and $\beta < 1$ represents sublinear scalability. While online iNMF was the fastest method on the tested data sizes, GLUE has a lower β, and could surpass online iNMF as data size increases to dozens of millions. **b**, Organ compositions in scRNA-seq and scATAC-seq. **c**, UMAP visualizations of the integrated cell embeddings showing only the scRNA-seq cells, colored by the original cell types. **d**, UMAP visualizations of the integrated cell embeddings showing only the scATAC-seq cells, colored by the original cell types. **e**, Alluvial diagram comparing the NNLS (non-negative least squares)-based and GLUE-based cell type annotations. The original NNLS-based annotations are to the left, and the GLUE-based annotations are to the right. Cells originally labeled as "Astrocytes" but mapped to "Excitatory neurons" are highlighted with pink flows. Cells originally labeled as "Astrocytes/Oligodendrocytes" but mapped to "Astrocytes" are highlighted with blue flows. Cells originally labeled as "Astrocytes/Oligodendrocytes" but mapped to "Oligodendrocytes" are highlighted with brown flows.

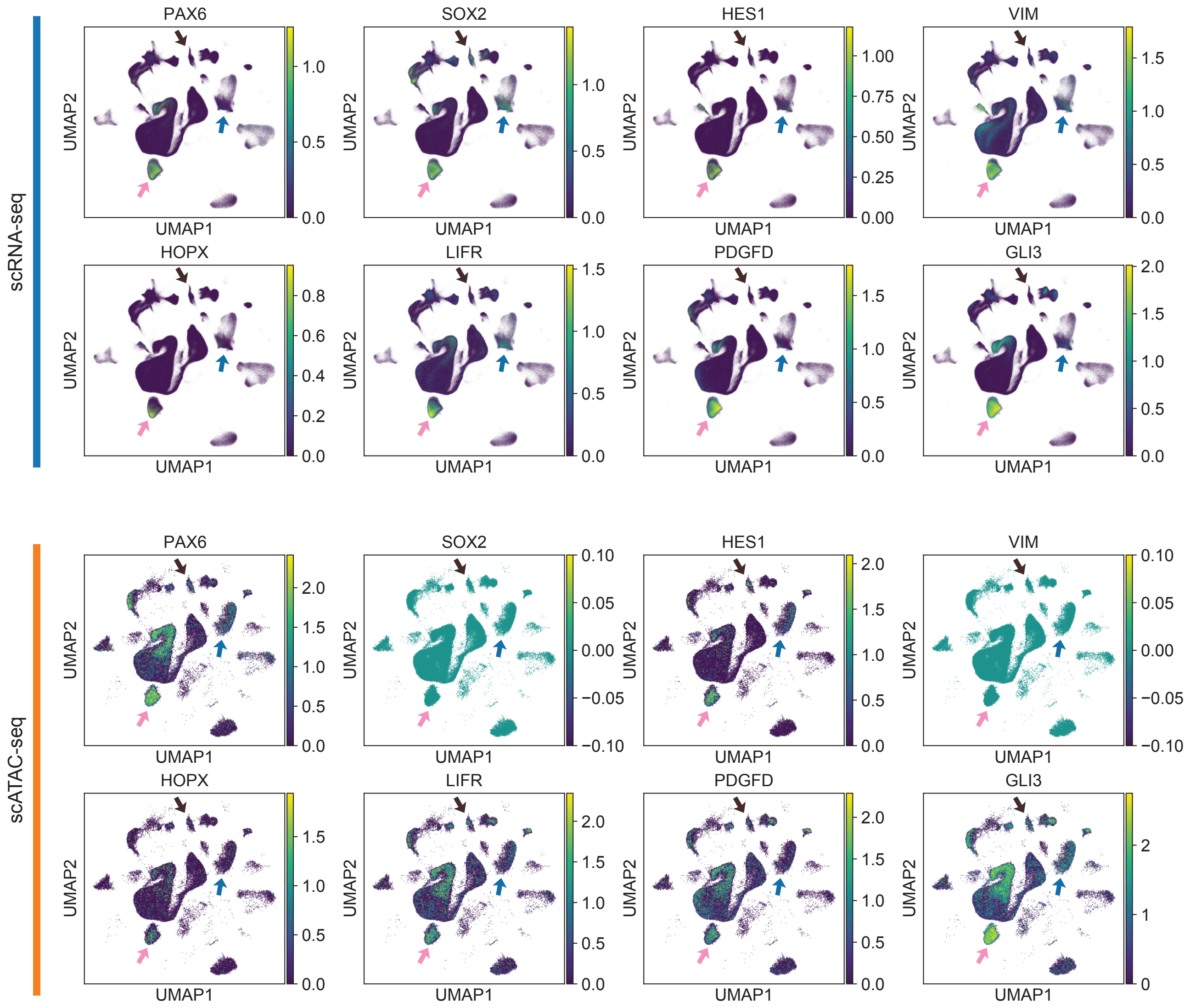

Supplementary Fig. 18 Gene expression and chromatin accessibility patterns of neural progenitor markers in cerebrum cells.
Putative neural progenitors are highlighted with pink arrows, astrocytes are highlighted with blue arrows, and oligodendrocytes are highlighted with brown arrows. *SOX2* and *VIM* were not detected in scATAC-seq, due to limitations in ATAC gene-level score calculation (no peaks overlap gene body and 2 kb upstream from TSS).

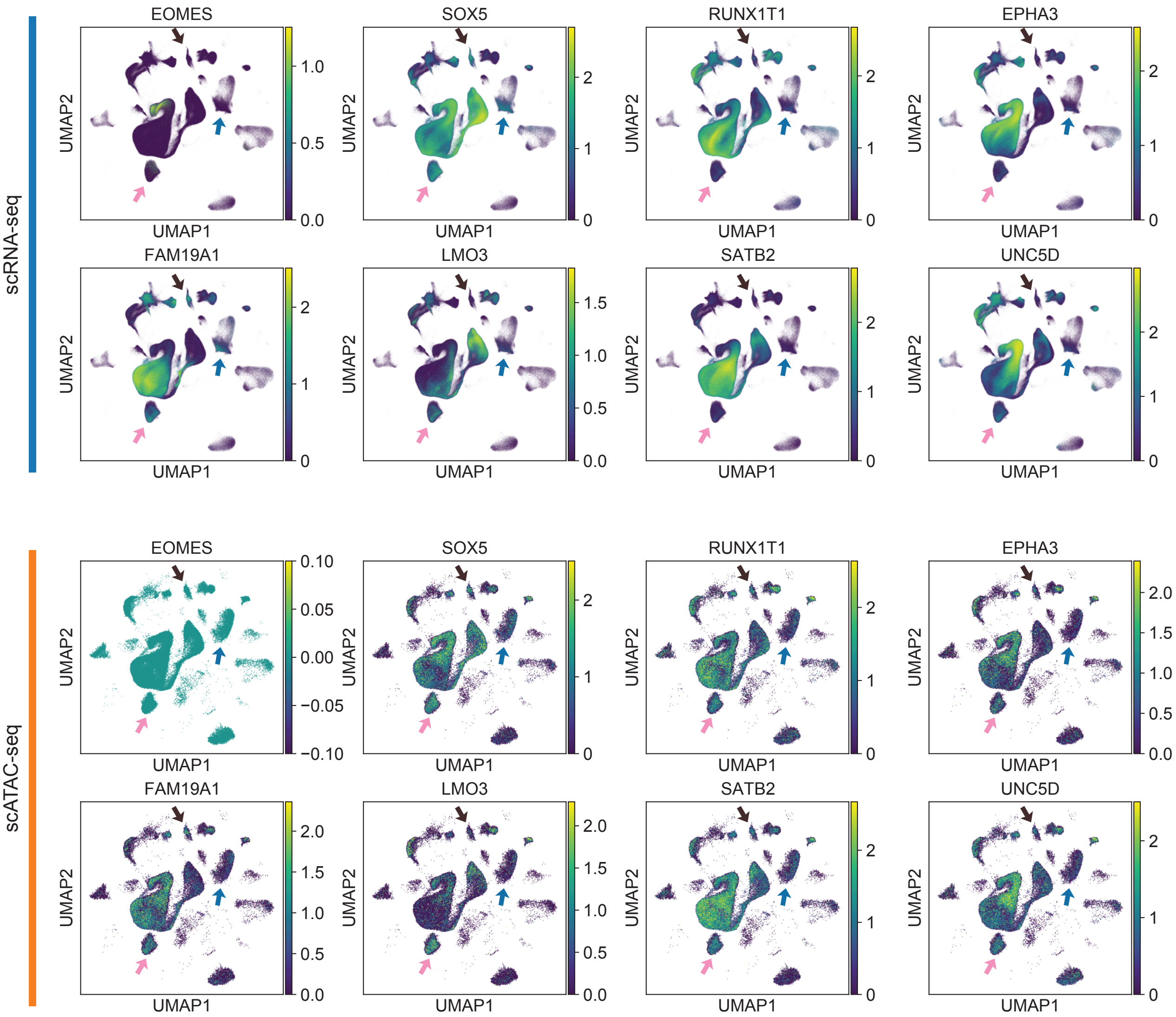

Supplementary Fig. 19 Gene expression and chromatin accessibility patterns of excitatory neuron markers in cerebrum cells.

Putative neural progenitors are highlighted with pink arrows, astrocytes are highlighted with blue arrows, and oligodendrocytes are highlighted with brown arrows. *EOMES* was not detected in scATAC-seq, due to limitations in ATAC gene-level score calculation (no peaks overlap gene body and 2 kb upstream from TSS).

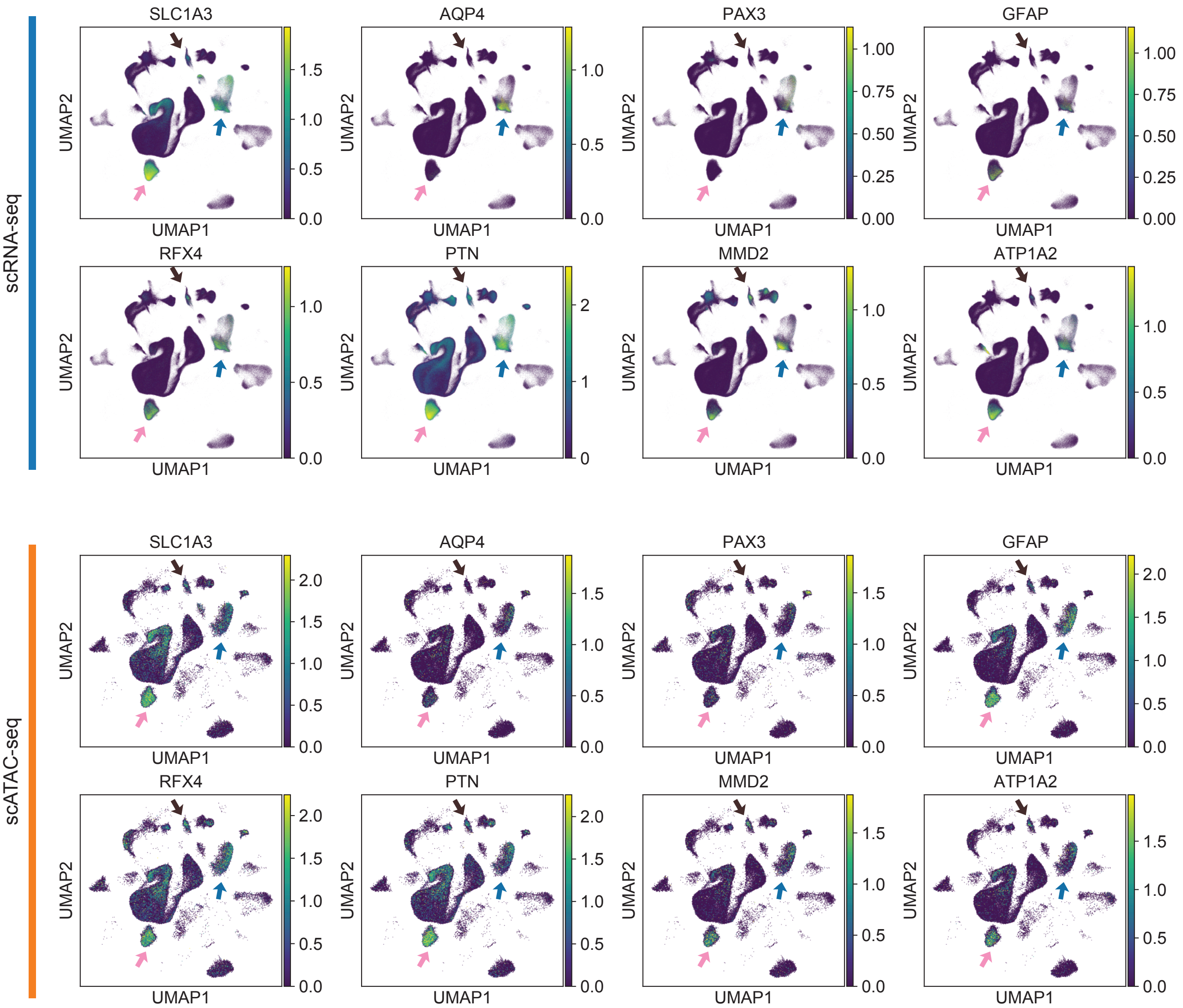

Supplementary Fig. 20 Gene expression and chromatin accessibility patterns of astrocyte markers in cerebrum cells.

Putative neural progenitors are highlighted with pink arrows, astrocytes are highlighted with blue arrows, and oligodendrocytes are highlighted with brown arrows.

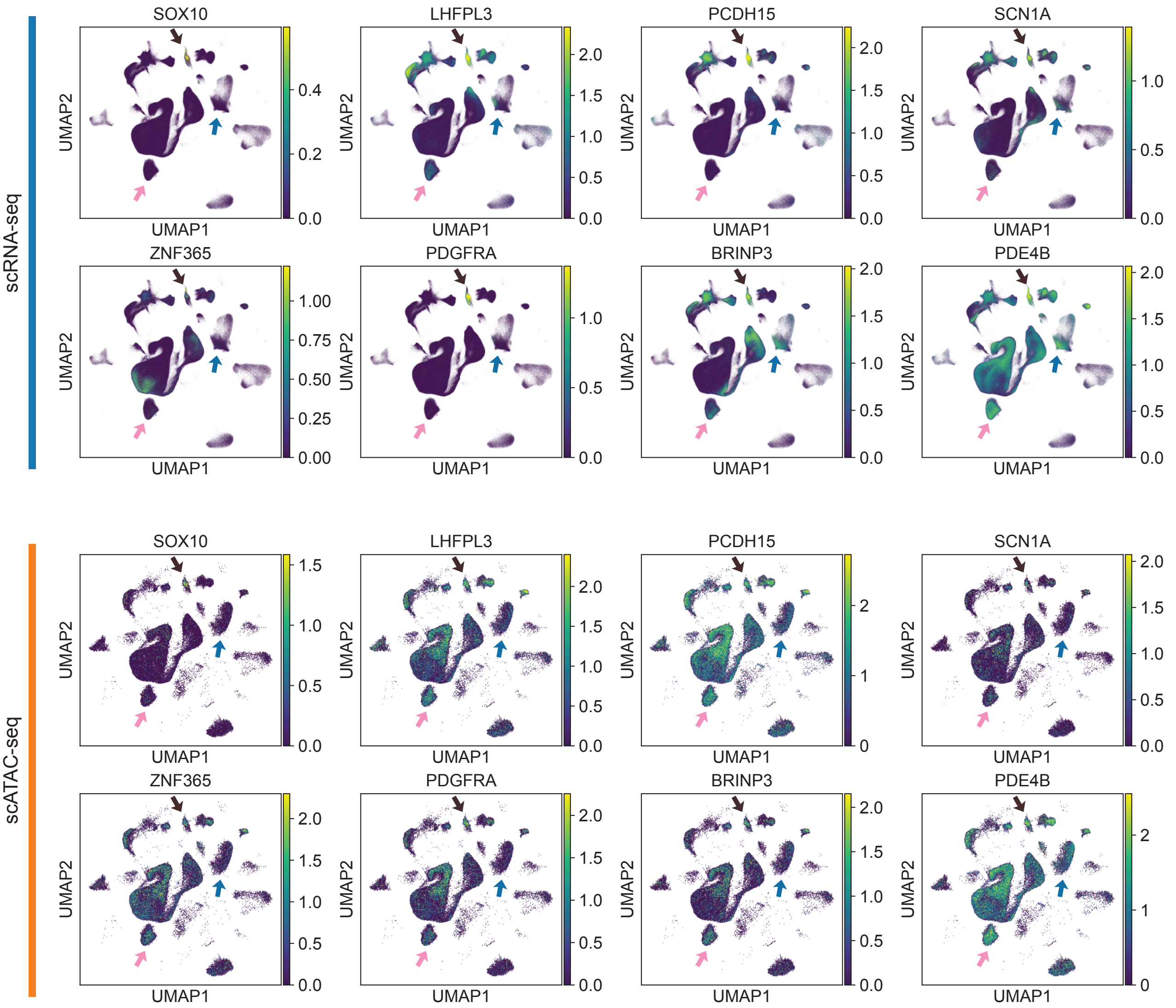

Supplementary Fig. 21 Gene expression and chromatin accessibility patterns of oligodendrocyte markers in cerebrum cells.

Putative neural progenitors are highlighted with pink arrows, astrocytes are highlighted with blue arrows, and oligodendrocytes are highlighted with brown arrows.

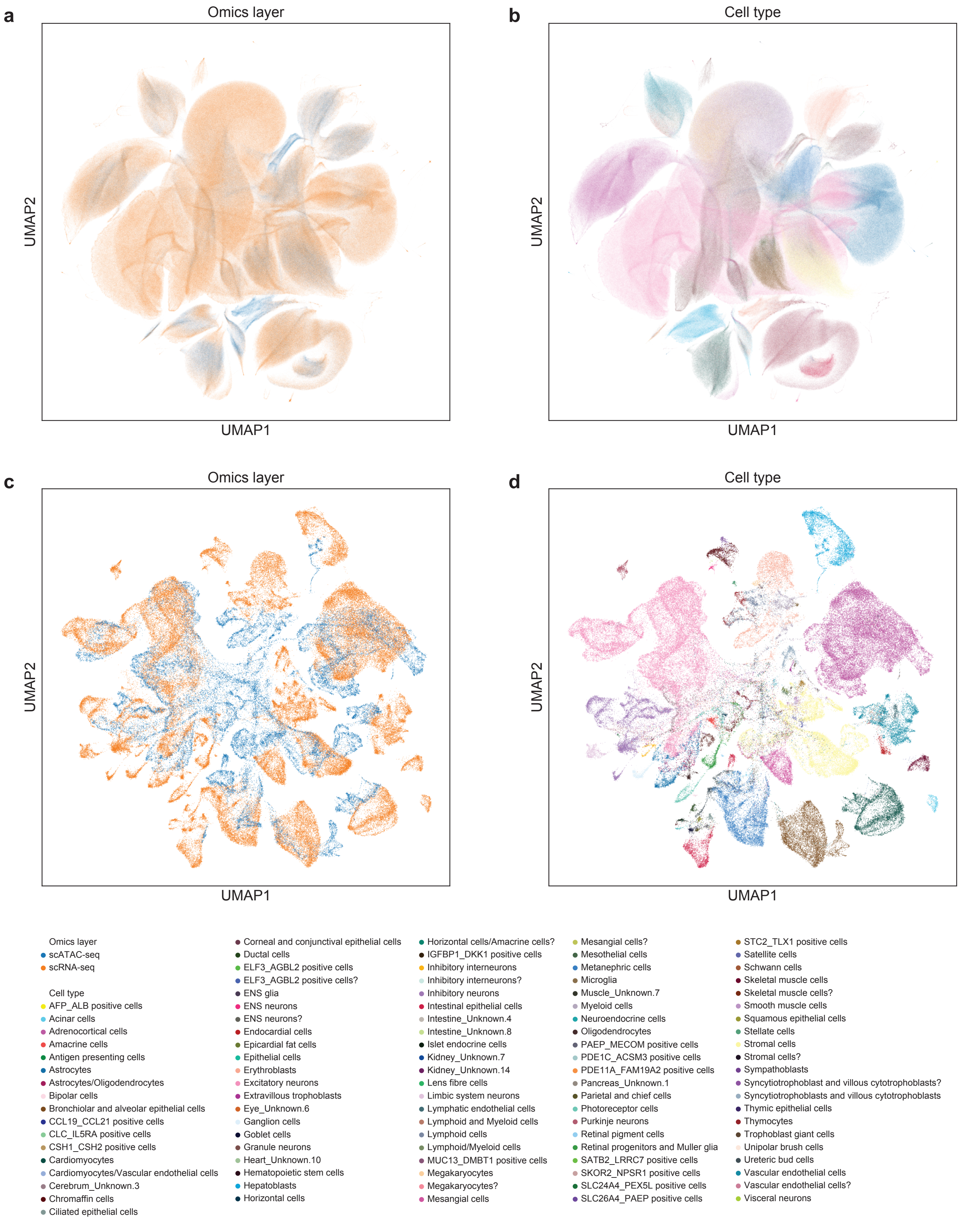

Supplementary Fig. 22 UMAP visualization of the multi-omics human cell atlas integrated by other methods.

a, **b**, Online iNMF-integrated cell embeddings colored by **a**, omics layers, and **b**, cell types. **c**, **d**, Seurat v3-integrated cell embeddings of aggregated metacells colored by **c**, omics layers, and **d**, cell types.

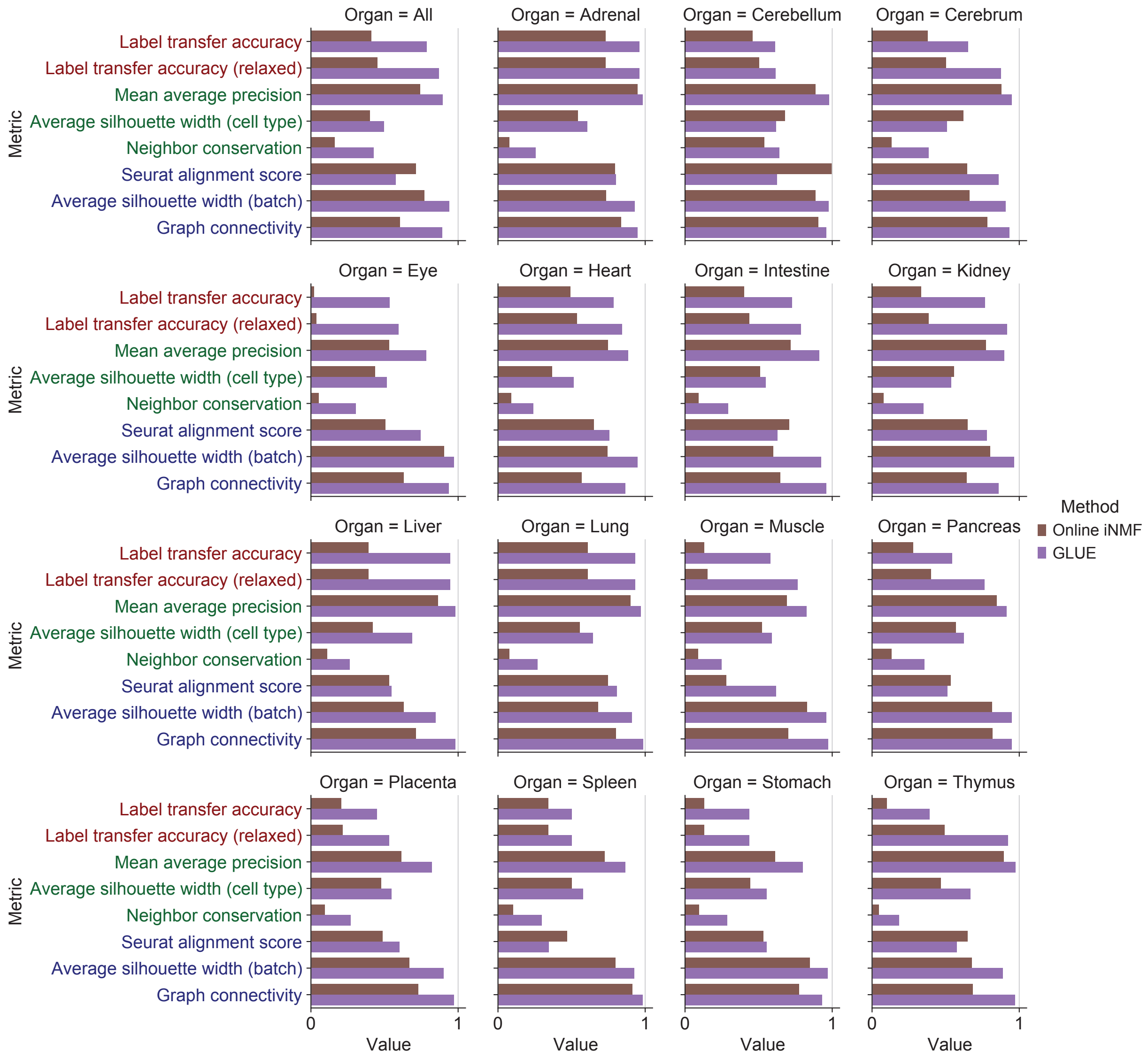

Supplementary Fig. 23 Performance metrics on the atlas integration.
Label-transfer based metrics are colored in red. Biology conservation metrics are colored in green. Omics mixing metrics are colored in blue.

Dataset	Protocol	Species	Organ	Cell number	Publication	PMID	Access code / URL
Chen-2019	SNARE-seq	Mus musculus	Cortex	9,190	High-throughput sequencing of the transcriptome and chromatin accessibility in the same cell	31611697	GSE126074
Ma-2020	SHARE-seq	Mus musculus	Skin	32,231	Chromatin potential identified by shared single-cell profiling of RNA and chromatin	33098772	GSE140203
10x-Multiome-Pbmc10k	10x Multiome	Homo sapiens	PBMC	9,631	NA	NA	https://support.10xgenomics.com/single-cell-multiome-atac-gex/datasets/1.0.0/pbmc_granulocyte_sorted_10k
Saunders-2018	Drop-seq	Mus musculus	Cortex	55,803	Molecular diversity and specializations among the cells of the adult mouse brain	30096299	http://dropviz.org/
Luo-2017	snmC-seq	Mus musculus	Cortex	3,377	Single-cell methylomes identify neuronal subtypes and regulatory elements in mammalian cortex	28798132	https://brainome.ucsd.edu/annoj/brain_single_nuclei/
10x-ATAC-Brain5k	10x ATAC	Mus musculus	Cortex	2,317	NA	NA	https://support.10xgenomics.com/single-cell-atac/datasets/1.1.0/atac_v1_adult_brain_fresh_5k
Cao-2020	sci-RNA-seq3	Homo sapiens	Whole-organism	4,062,980	A human cell atlas of fetal gene expression	33184181	GSE156793
Domcke-2020	sci-ATAC-seq3	Homo sapiens	Whole-organism	720,613	A human cell atlas of fetal chromatin accessibility	33184180	GSE149683
Muto-2021	snRNA-seq, snATAC-seq	Homo sapiens	Kidney	44,190	Single cell transcriptional and chromatin accessibility profiling redefine cellular heterogeneity in the adult human kidney	33850129	GSE151302
Yao-2021	scRNA 10x v3, snATAC-seq	Mus musculus	MOp	124,571	A transcriptomic and epigenomic cell atlas of the mouse primary motor cortex	34616066	https://assets.nemoarchive.org/dat-ch1nqb7

Supplementary Table 1 Public datasets used in the study.
