# Supplementary material for: Multi-omics single-cell data integration and regulatory inference with graph-linked embedding
Source: Nat Biotechnol. 2022 May 2;40(10):1458–66. doi: 10.1038/s41587-022-01284-4 (PMC9546775; doi:10.1038/s41587-022-01284-4)
Supplement: Supplementary file 2 — Reporting Summary [file 41587_2022_1284_MOESM2_ESM.pdf]

nature research

Corresponding author(s): Ge Gao

Last updated by author(s): Mar 12, 2022

Reporting Summary

Nature Research wishes to improve the reproducibility of the work that we publish. This form provides structure for consistency and transparency in reporting. For further information on Nature Research policies, see our Editorial Policies and the Editorial Policy Checklist.

Statistics

For all statistical analyses, confirm that the following items are present in the figure legend, table legend, main text, or Methods section.

n/a	Confirmed	
☐	☒	The exact sample size (*n*) for each experimental group/condition, given as a discrete number and unit of measurement
☐	☒	A statement on whether measurements were taken from distinct samples or whether the same sample was measured repeatedly
☐	☒	The statistical test(s) used AND whether they are one- or two-sided<br>*Only common tests should be described solely by name; describe more complex techniques in the Methods section.*
☐	☒	A description of all covariates tested
☐	☒	A description of any assumptions or corrections, such as tests of normality and adjustment for multiple comparisons
☐	☒	A full description of the statistical parameters including central tendency (e.g. means) or other basic estimates (e.g. regression coefficient) AND variation (e.g. standard deviation) or associated estimates of uncertainty (e.g. confidence intervals)
☐	☒	For null hypothesis testing, the test statistic (e.g. *F*, *t*, *r*) with confidence intervals, effect sizes, degrees of freedom and *P* value noted<br>*Give P values as exact values whenever suitable.*
☒	☐	For Bayesian analysis, information on the choice of priors and Markov chain Monte Carlo settings
☒	☐	For hierarchical and complex designs, identification of the appropriate level for tests and full reporting of outcomes
☐	☒	Estimates of effect sizes (e.g. Cohen's *d*, Pearson's *r*), indicating how they were calculated

Our web collection on statistics for biologists contains articles on many of the points above.

Software and code

Policy information about availability of computer code

Data collection	Data collection was performed using custom code available at https://github.com/gao-lab/GLUE/tree/master/data/collect.
Data analysis	GLUE analysis was performed using custom Python (v3.8.5) package scglue (v0.2.0) (available at https://github.com/gao-lab/GLUE). Benchmarked methods were performed using software packages unioncom (v0.3.0), Pamona (v0.1.0), rliger (v1.0.0), harmony (v0.1.0), bindSC (v1.0.0), Seurat (v4.0.2). Benchmarking pipeline was implemented using Snakemake (v6.12.3). Full environment configuration files can also be found at https://github.com/gao-lab/GLUE.

For manuscripts utilizing custom algorithms or software that are central to the research but not yet described in published literature, software must be made available to editors and reviewers. We strongly encourage code deposition in a community repository (e.g. GitHub). See the Nature Research guidelines for submitting code & software for further information.

Data

Policy information about availability of data

All manuscripts must include a data availability statement. This statement should provide the following information, where applicable:

- Accession codes, unique identifiers, or web links for publicly available datasets
- A list of figures that have associated raw data
- A description of any restrictions on data availability

All datasets used in this study are already published and were obtained from public data repositories. See Supplementary Table 1 for detailed information on single-cell omics datasets used in this study, including access codes and URLs. For regulatory inference and evaluation, the pcHi-C data was obtained from supplementary file of the original publication (https://www.sciencedirect.com/science/article/pii/S0092867416313228), eQTL data from GTEx v8 (https://www.gtexportal.org/home/datasets), TF ChIP-seq data from ENCODE data portal (https://www.encodeproject.org/), and TRRUST v2 database from the official website (https://www.grnpedia.org/trrust/downloadnetwork.php). All benchmarking source data are available in Supplementary Data 1.

Field-specific reporting

Please select the one below that is the best fit for your research. If you are not sure, read the appropriate sections before making your selection.

☒ Life sciences ☐ Behavioural & social sciences ☐ Ecological, evolutionary & environmental sciences

For a reference copy of the document with all sections, see nature.com/documents/nr-reporting-summary-flat.pdf

Life sciences study design

All studies must disclose on these points even when the disclosure is negative.

Sample size	All computational evaluations were repeated multiple times using different random initializations to evaluate algorithmic stability. For most evaluations, we used n=8 repeats which was empirically sufficient to capture the mean and variance of model performance. The only exception was hyperparameter evaluation, where we used n=4 repeats (a minimal for empirical estimation of mean and variance) to reduce computational cost due to the large number of hyperparameter combinations evaluated.
Data exclusions	No data were excluded from analysis.
Replication	All computational experiments were assembled using Snakemake and environment configuration files are provided to ensure reproducibility. All attempts at replication were successful.
Randomization	Complete randomization was used for corruption and subsampling evaluations.
Blinding	All computational methods were blinded to ground truth cell type labels/pairing information during performance evaluation.

Reporting for specific materials, systems and methods

We require information from authors about some types of materials, experimental systems and methods used in many studies. Here, indicate whether each material, system or method listed is relevant to your study. If you are not sure if a list item applies to your research, read the appropriate section before selecting a response.

Materials & experimental systems

n/a	Involved in the study	
☒	☐	Antibodies
☒	☐	Eukaryotic cell lines
☒	☐	Palaeontology and archaeology
☒	☐	Animals and other organisms
☒	☐	Human research participants
☒	☐	Clinical data
☒	☐	Dual use research of concern

Methods

n/a	Involved in the study	
☒	☐	ChIP-seq
☒	☐	Flow cytometry
☒	☐	MRI-based neuroimaging
